# The MAO inhibitors phenelzine and clorgyline revert enzalutamide resistance in castration resistant prostate cancer

**DOI:** 10.1038/s41467-020-15396-5

**Published:** 2020-06-01

**Authors:** Keliang Wang, Jie Luo, Shuyuan Yeh, Bosen You, Jialin Meng, Philip Chang, Yuanjie Niu, Gonghui Li, Changxue Lu, Yezi Zhu, Emmanuel S. Antonarakis, Jun Luo, Chi-Ping Huang, Wanhai Xu, Chawnshang Chang

**Affiliations:** 1 Department of Urology, The 4th Affiliated Hospital of Harbin Medical University, NHC Key Lab of Molecular Probes and Targeted Diagnosis and Therapy, Harbin, 150001 China; 2 George Whipple Lab for Cancer Research, Departments of Pathology, Urology, Radiation Oncology, and The Wilmot Cancer Institute, University of Rochester Medical Center, Rochester, NY 14642 USA; 3 Department of Neurology, Kaiser Permanente Santa Clara Medical Center, Santa Clara, CA 95051 USA; 4 Tianjin Institute of Urology, Tianjin Medical University, Tianjin, 300211 China; 5 Department of Urology, Sir Run Run Shaw Hospital, Zhejiang University School of Medicine, Hangzhou, 310016 China; 6 Prostate Cancer Program, Sidney Kimmel Comprehensive Cancer Center, and James Buchanan Brady Department of Urology, Johns Hopkins University School of Medicine, Baltimore, MD 21287 USA; 7 Sex Hormone Research Center, Department of Urology, China Medical University and Hospital, Taichung, 404 Taiwan

**Keywords:** Urological cancer, Prostate cancer

## Abstract

The antiandrogen enzalutamide (Enz) has improved survival in castration resistant prostate cancer (CRPC) patients. However, most patients eventually develop Enz resistance that may involve inducing the androgen receptor (AR) splicing variant 7 (ARv7). Here we report that high expression of monoamine oxidase-A (MAO-A) is associated with positive ARv7 detection in CRPC patients following Enz treatment. Targeting MAO-A with phenelzine or clorgyline, the FDA-approved drugs for antidepression, resensitize the Enz resistant (EnzR) cells to Enz treatment and further suppress EnzR cell growth in vitro and in vivo. Our findings suggest that Enz-increased ARv7 expression can transcriptionally enhance MAO-A expression resulting in Enz resistance via altering the hypoxia HIF-1α signals. Together, our results show that targeting the Enz/ARv7/MAO-A signaling with the antidepressants phenelzine or clorgyline can restore Enz sensitivity to suppress EnzR cell growth, which may indicate that these antidepression drugs can overcome the Enz resistance to further suppress the EnzR CRPC.

## Abstract (web summary)

Castration resistant prostate cancer patients treated with enzalutamide may develop resistance to the drug. Here, the authors report that monoamine oxidase-A expression is increased in these resistant tumors and that the antidepressants phenelzine/clorgyline can reverse such resistance to further suppress tumor growth

## Introduction

Prostate cancer (PCa) remains one of the most prevalent male cancers in the United States, with over an estimated 26,000 cancer deaths annually^1,2^. Presently, androgen deprivation therapy (ADT) with antiandrogens to suppress androgen synthesis or prevent androgens from binding to the androgen receptor (AR) are the standard treatments for metastatic PCa^3,4^. Unfortunately, most ADT, including the recently developed potent antiandrogen enzalutamide (Enz), eventually fails. As a result, castration resistance, including Enz resistance, represents a lethal disease stage with limited management options^3,4^.

Recent studies indicated that the Enz resistance may involve the induction of AR variants^5^ or AR-F876L mutant expression^6,7^, overexpression of glucocorticoid receptor (GR)^8,9^, activation of the IL6/Stat3/AR axis^10^, or NFkB2/p52 axis^11^. However, only the induction of the AR variant 7 (ARv7), a constitutively active AR variant lacking the C-terminal ligand-binding domain^12,13^, has the strong clinical data support^14^. Detection of ARv7 in metastatic CRPC patients often indicates lack of response to Enz treatment^15^. The detailed mechanisms underlying the role of Enz-induced ARv7 and approaches to antagonize this resistance mechanism, however, remain unclear.

Monoamine oxidase-A (MAO-A) is the key enzyme catalyzing the deamination of amines and may play key roles in degrading neurotransmitters^16^, including norepinephrine, dopamine, and serotonin^17^. Results from clinical studies revealed that MAO might play key roles for the progression of some neuron disorders, including depression^18^, Parkinson’s disease^19^, or Alzheimer’s disease^20^. Other studies indicated that MAO-A is also involved in tumor progression by altering cell proliferation and apoptosis^21,22^, or by modulating the epithelial–mesenchymal transition signals^23,24^. Clorgyline, a selective MAO-A inhibitor for antidepression, was shown to suppress PCa progression in the mouse model^25^, and phenelzine, another MAO-A inhibitor, the FDA-approved drug for antidepression, is under phase II clinical trials for nonmetastatic recurrent PCa (ClinicalTrials.gov Identifier: NCT02217709). The connection of MAO-A to the development of Enz resistance in CRPC, however, has not been investigated.

Here, we unexpectedly found that MAO-A is highly expressed in several Enz-resistant (EnzR) PCa cells, and its expression is mediated by ARv7. Targeting MAO-A with its specific inhibitor clorgyline^26^ or phenelzine^27^, the existing antidepression drugs, can restore Enz sensitivity to further suppress EnzR cell growth. We provide preclinical data supporting the clinical development of these existing FDA-approved drugs for the purpose of treating mCRPC patients with elevated ARv7.

## Results

### Increased MAO-A expression in the Enz resistant cells

The antiandrogen Enz is clinically effective in treating CRPC patients. However, the development of Enz resistance is inevitable, emphasizing the need to further dissect the mechanisms of Enz resistance in basic preclinical studies using in vitro cell lines and in vivo mouse models^14^.

We first searched for potential altered genes after the development of Enz resistance in EnzR CRPC cells generated after chronic culture of CRPC C4-2 cells in media containing increasing Enz concentrations from 10 to 30 μM for 1 year (named as EnzR1-C4-2), or after continuously culturing the C4-2 cells in media containing fixed 10 μM Enz for 6 months (named as EnzR2-C4-2), We also used the naturally EnzR cell line, CWR22Rv1, and named them as EnzR3-22Rv1. Finally, we also obtained the EnzR C4-2B cells from Dr. Allen Gao and named them as EnzR4-C4-2B in these studies.

**Fig. 1:**
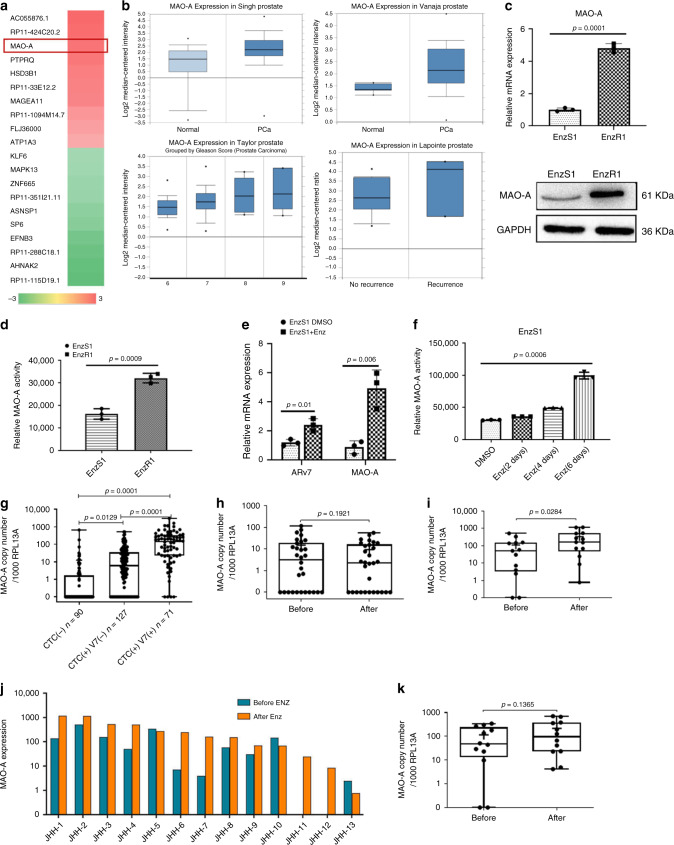
MAO-A expression is associated with the development of Enz resistance. **a** Heat map of most significantly changed genes in EnzR1-C4-2 cells. **b** Statistical analysis of MAO-A expression in published datasets. The expressions of MAO-A in normal prostate tissues and PCa tissues were analyzed based on Singh (normal, *n* = 50; cancer, *n* = 52) and Vanaja (normal, *n* = 8; cancer, *n* = 32,) datasets. The MAO-A expressions in different Gleason score PCa samples were analyzed based on the Taylor dataset (Gleason score 6, *n* = 79; Gleason score 7, *n* = 50; Gleason score 8, *n* = 10; and Gleason score 9, *n* = 9). The MAO-A expression in PCa samples with recurrence and no recurrence were analyzed based on Lapointe (no recurrence, *n* = 14; recurrence, *n* = 3) dataset. The boxes extend from the 25th to 75th percentiles with the median value plotted at the middle line. The maximum and minimum values were indicated with points and labeled on the figures. **c** The qPCR and western blot analysis of MAO-A levels in EnzR1-C4-2 and EnzS1-C4-2 cells; (*n* = 3 biological independent samples for qPCR). **d** MAO-A activity analysis in EnzS1-C4-2 and EnzR1-C4-2 cells by using MAO-Glo assay; (*n* = 3 biological independent samples). **e** MAO-A and ARv7 mRNA levels by qPCR following treatment with DMSO or 10 μM Enz in EnzS1-C4-2 cells for 6 days; (*n* = 3 biological independent samples). **f** MAO-A activity analyzed in EnzS1-C4-2 cells treated with 10 μM Enz for 6 days; (*n* = 3 biological independent samples). **g** Absolute copy numbers of MAO-A mRNA normalized to copy number of RPL13A in CTCs isolations from 288 patients. Those samples were grouped by CTC and ARv7 status: CTC−, CTC+/ARv7−, and CTC+/ARv7+. **h** MAO-A copy numbers normalized by RPL13A in patients whose ARv7 status remained negative after Enz treatment (*N* = 30). **i** MAO-A copy numbers normalized by RPL13A in patients whose ARv7 status changed from negative to positive after Enz treatment (*N* = 13). **j**, **k** Relative MAO-A copy number values at baseline (before Enz) and after Enz treatment (after Enz) in each patient whose ARv7 status changed from negative to positive after Enz treatment (*N* = 13) **j**, and in patients whose ARv7 status was positive at baseline (before Enz) and remained positive after Enz treatment (*N* = 12) **k**. For **g**–**i**, **k**: relative MAO-A expression values within groups were shown, in which the box extended from the 25th to 75th percentiles with the median value plotted at the middle line, with all data points shown from minimal to maximal values indicated with whiskers. Data represent the mean ± SEM, error bars represent SEM. *p*-value was determined by two-tailed paired *t*-test.

We then applied RNAseq assay to compare the expression profiles in EnzR1-C4-2 cells and the C4-2 parental Enz-sensitive (EnzS1-C4-2) cells. Among many genes that were highly differentially expressed in EnzR1-C4-2 cells, MAO-A expression was increased significantly in the EnzR1-C4-2 cells by more than six folds (Fig. 1a). Since recent studies indicated that the expression of MAO-A is correlated with the poorly differentiated human PCa^23,24^, we analyzed MAO-A expression in four different human PCa datasets. The results revealed higher expression of MAO-A in human PCa tissues compared to normal prostate tissues (Fig. 1b), and higher MAO-A expression in higher Gleason score tumors and recurrent PCa (Fig. 1b, Supplementary Fig. 1a).

We further validated the RNAseq data, and found MAO-A (mRNA and protein) expression were significantly increased in the EnzR1-C4-2 cells as compared to the EnzS1-C4-2 cells (Fig. 1c). In addition, MAO-A activity was higher in EnzR1-C4-2 cells than EnzS1-C4-2 cells by MAO-GLO assay (Fig. 1d). Similar results were also obtained when we replaced another paired EnzR vs EnzS cell line (EnzR4-C4-2B vs EnzS4-C4-2B cells; Supplementary Fig. 1b).

Importantly, we found that treating the EnzS1-C4-2 cells with 10 μM Enz for 6 days could increase both MAO-A and ARv7 mRNA expressions (Fig. 1e), suggesting that Enz may increase MAO-A expression by activating key transcription factors, such as ARv7. Similar results were obtained when we treated EnzS5-VCaP cells with 10 µM for 10 days (Supplementary Fig. 1c). Results from MAO-GLO assay also confirmed that adding 10 μM Enz led to increase MAO-A activity in EnzS1-C4-2 cells (Fig. 1f).

Therefore, similar to an increase of ARv7 expression in cells treated with Enz^14,28^, an increase in MAO-A expression in Enz-treated cells may also play a role in mediating the Enz resistance.

### Elevated MAO-A level in CTCs from patients treated with Enz

To further corroborate the in vitro cell lines data, we evaluated MAO-A expression in a total of 288 blood circulating tumor cells (CTCs) samples collected from men with mCRPC undergoing treatment with standard-of-care systemic therapies. These 288 blood samples were processed for CTCs and then cDNA and finally were divided into three biomarker groups^29^: (1) CTC negative (CTC−) (*n* = 90); (2) CTC positive (CTC+)/ARv7− (*n* = 127); and (3) CTC+/ARv7 + (*n* = 71). We found significantly increased MAO-A mRNA in ARv7+ CTCs compared to ARv7− CTCs (Fig. 1g), suggesting a positive correlation between ARv7 and MAO-A expression.

Subseqeunt analysis focused on patients treated with Enz with paired samples collected at treatment baseline and at disease progression. Among the 288 samples, there were 30 pairs of samples (*n* = 60) from those that were negative for ARv7 at baseline and remained negative at the time of progression on Enz (ARv7− to ARv7− group), 13 pairs of samples (*n* = 26) from those with a conversion from ARv7− status to ARv7+ status (ARv7− to ARv7+ group), and 12 pairs of samples (*n* = 24) from those who were positive for ARv7 at baseline and remained positive at the time of progression (ARv7+ to ARv7+ group). In those CTCs that remained ARv7− after Enz treatment, MAO-A expression showed little difference (Fig. 1h). In contrast, in those CRPC patients whose ARv7 expression changed from negative to positive after Enz treatment, the MAO-A expression levels increased dramatically, suggesting that MAO-A expression may only increase in those ARv7+ patients (Fig. 1i, j). In CRPC patients with ARv7+ at baseline and remained positive after Enz treatment, there was no further increase of MAO-A expression after Enz treatment, MAO-A expression trended higher after treatment with Enz, but the difference was not significant (Fig. 1k). This is consistent with the lack of further ARv7 increase after Enz treatment, if ARv7 was detected at the baseline^14^. We did not find statistically significant differences for the full-length AR (AR-FL) comparing pretreatment and posttreatment samples in any of the paired groups (Supplementary Fig. 2a–c), suggesting that elevated MAO-A expression following the treatment with Enz may be mediated by the ARv7.

Together, results from human CRPC patients’ CTC analysis (Fig. 1g–k) demonstrate that MAO-A expression is positively associated with ARv7 expression in CRPC patients treated with Enz. Together with our in vitro cell lines data (Fig. 1c, d), these human clinical findings suggest that Enz can increase both ARv7 and MAO-A expression, and Enz-induced MAO-A expression may depend on the ARv7 status.

### Phenelzine or clorgyline can revert Enz resistance

**Fig. 2:**
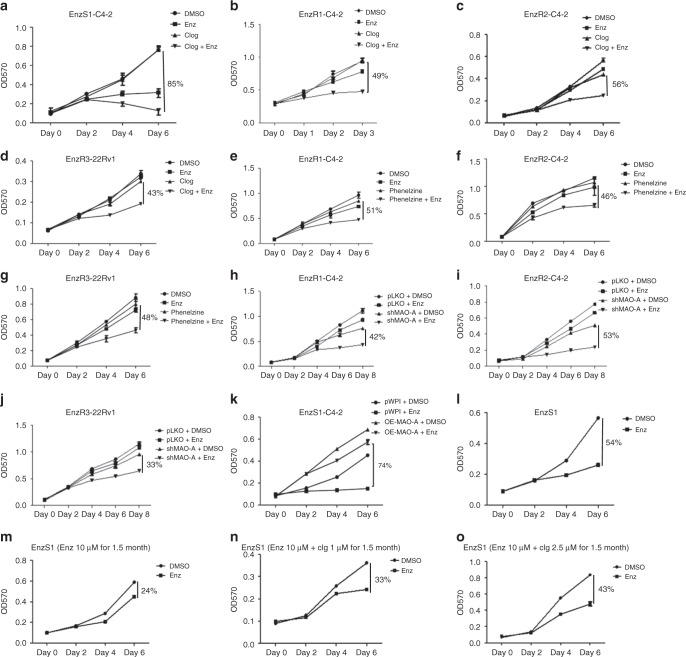
Targeting MAO-A resensitizes EnzR cells to Enz and suppresses EnzR cell growth. **a** EnzS1-C4-2 cells were treated with/without (w/o) 10 μM Enz and 5 μM clorgyline, and cell viability analyzed by MTT assay; (*n* = 3 biological independent samples); *p* = 0.03. **b**–**d** EnzR1-C4-2, EnzR2-C4-2, and EnzR3-22Rv1 cells, respectively, were treated (w/o) 10 μM Enz and 5 μM clorgyline, and cell viability was analyzed by MTT assay; (*n* = 3 biological independent samples); *p* = 0.02 for **b**, *p* = 0.03 for **c**, and *p* = 0.03 for **d**. **e**–**g** EnzR1-C4-2, EnzR2-C4-2, and EnzR3-22Rv1 cells, respectively, were treated w/o 10 μM Enz and 5 μM phenelzine, and cell viability was analyzed; (*n* = 3 biological independent samples); *p* = 0.03 for **e**, *p* = 0.04 for **f**, and *p* = 0.03 for **g**. **h**–**j** The EnzR1-C4-2, EnzR2-C4-2, and EnzR3-22Rv1 cells with pLKO or shMAO-A cells were treated w/o Enz and cell viability was analyzed; (*n* = 3 biological independent samples); *p* = 0.02 for **h**, *p* = 0.02 for **i**, and *p* = 0.01 for **j**. **k** MAO-A was overexpressed (oeMAO-A) in EnzS1-C4-2 cells and then the cells treated w/o Enz and cell viability was analyzed; (*n* = 3 biological independent samples); *p* = 0.002 for **k**. **l** EnzS1-C4-2 cells were treated w/o 10 μM Enz and cell viability was analyzed; (*n* = 3 biological independent samples); *p* = 0.001. **m** EnzS1-C4-2 cells were first treated (w/o) 10 μM Enz for 1.5 months. And then the cells were seeding and the cell viability under 10 μM Enz treatment was analyzed by MTT assay; (*n* = 3 biological independent samples); *p* = 0.02. **n**, **o** EnzS1-C4-2 cells were treated w/o 10 μM Enz and 1 μM (**n**) or 2.5 μM (**o**) clorgyline (clg) for 1.5 months. And then the cell viability under 10 μM Enz treatment was analyzed; (*n* = 3 biological independent samples); *p* = 0.01 for **n** and *p* = 0.01 for **o**. Data represent the mean ± SEM, error bars represent SEM. *p*-value was determined by two-tailed paired *t*-test.

To further link the increased MAO-A expression to the development of Enz resistance in CRPC cells, we treated the CRPC cells with a selective MAO-A inhibitor, clorgyline, used as antidepression drug^27^. Results from MTT proliferation assay revealed that while clorgyline treatment alone failed to suppress EnzS1-C4-2 cell proliferation, the combination of clorgyline and Enz treatment significantly inhibited (85%) EnzS1-C4-2 cell growth at day 6 (Fig. 2a). Importantly, EnzR1-C4-2 cell proliferation was also suppressed by adding clorgyline to Enz treatment (49% suppression), suggesting restoration of Enz sensitivity (Fig. 2b). Similar results were obtained in EnzR2-C4-2 cells (56% suppression; Fig. 2c) or EnzR3-22RV1 (43% suppression) cells (Fig. 2d).

We repeated the same set of experiments with the FDA-approved MAO-A inhibitor, phenelzine (10 μM) used to treat depression^19^, and results also revealed that suppressing the MAO-A activity could resensitize EnzR1-C4-2 (51% suppression), EnzR2-C4-2 (46% suppression), and EnzR3-22RV1 cells (48% suppression) to Enz treatment (Fig. 2e–g).

We then applied the gene expression perturbation approach using MAO-A-shRNA to suppress MAO-A, and results revealed that targeting MAO-A with MAO-A-shRNA also significantly increased Enz sensitivity and suppressed EnzR cells proliferation (Fig. 2h–j), with suppression rates of 42% in EnzR1-C4-2, 35% in EnzR2-C4-2, and 28% in EnzR3-22Rv1 cells. In contrast, overexpressing MAO-A in EnzS1-C4-2 cells resulted in Enz resistance (Fig. 2k).

Together, results from Fig. 2a–k using multiple EnzR CRPC cells with various inhibitors/blockers to either suppress MAO-A activity or suppress MAO-A expression all demonstrate that Enz-induced MAO-A expression contributed to the development of Enz resistance, and suppressing the MAO-A activity with antidepression drugs clorgyline or phenelzine (or MAO-A-shRNA to suppress its expression) all led to restore Enz sensitivity to further suppress EnzR cell proliferation.

### Clorgyline delays the development of Enz resistance

In addition to suppressing the EnzR cell growth, we are interested to see if clorgyline can also delay the development of Enz resistance in CRPC cells. We first treated EnzS1-C4-2 cells with 10 μM Enz alone or combined 10 μM Enz with 1 μM or 2.5 μM clorgyline for 1.5 months. We then treated the cells with Enz and evaluated the cell proliferation by MTT assays on days 0, 2, 4, and 6. The results revealed that while 1.5 months of Enz treatment alone could induce the development of Enz resistance by reducing Enz sensitivity from 54% to 24%.(Fig. 2l vs m), the combination of Enz and clorgyline delayed the development of Enz resistance by increasing Enz sensitivity from 54%→24% to 54%→33% (at 1 μM clorgyline, Fig. 2m vs n) or from 54%→24% to 54%→43% (at 2.5 μM clorgyline, Fig. 2m vs o).

Together, these results (Fig. 2l–o) suggest that targeting MAO-A with the inhibitor clorgyline can also delay the development of Enz resistance.

### Enz induces MAO-A level via increasing ARv7 expression

**Fig. 3:**
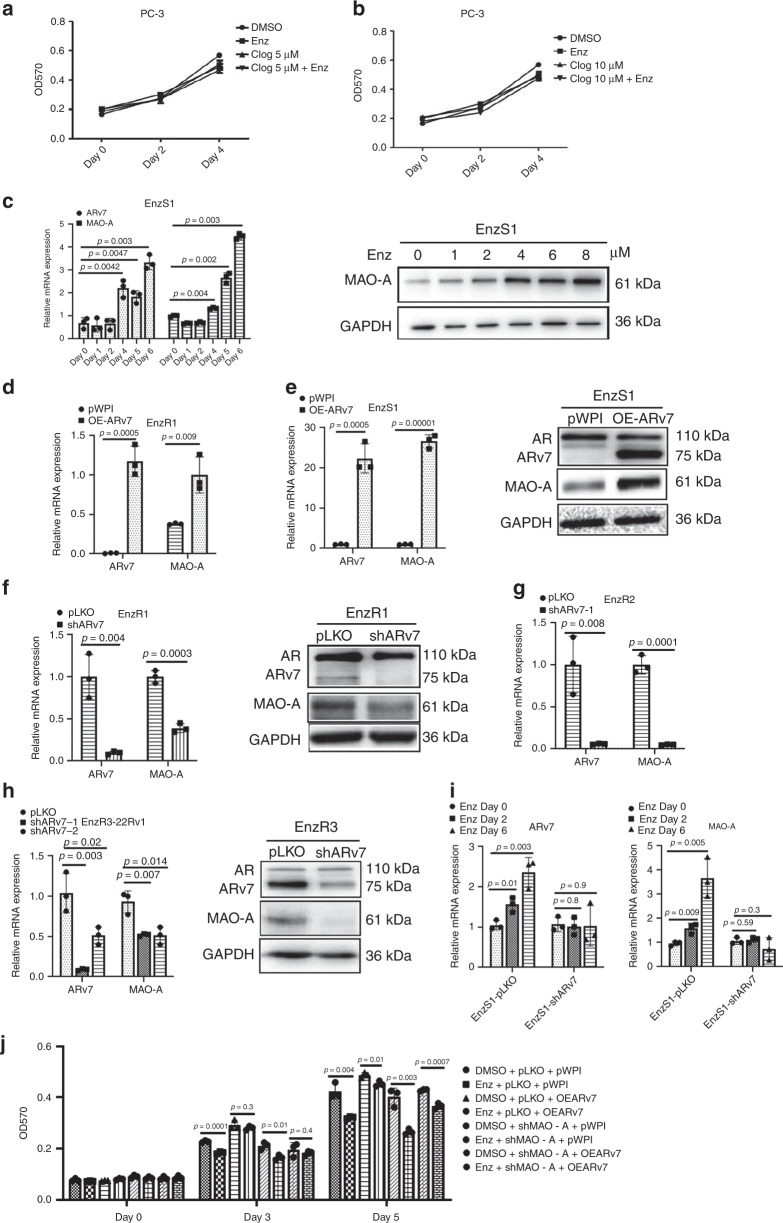
Mechanism dissection of how Enz increases the MAO-A expression: via increasing ARv7. **a**, **b** PC3 cells were treated with/without (w/o) 10 μM Enz and 5 μM clorgyline or 10 μM clorgyline. The cell viability was analyzed by MTT assay; (*n* = 3 biological independent samples). **c** EnzS1-C4-2 cells were treated with 10 μM Enz for different time points. The ARv7 and MAO-A mRNA level were analyzed by qPCR (left) and western blot (right); (*n* = 3 biological independent samples). **d** The mRNA levels of ARv7 and MAO-A were analyzed in EnzR1-C4-2 pWPI and pWPI-ARv7 cells. **e** The expression of ARv7 and MAO-A were analyzed in EnzS1-C4-2 pWPI and pWPI-ARv7 (oeARv7) cells by qPCR (left) and western blot (right). **f**–**h** The mRNA and protein level of ARv7 and MAO-A were analyzed in EnzR1-C4-2, EnzR2-C4-2 (no protein shown), EnzR3-22Rv1 pLKO, and shARv7 cells; (*n* = 3 biological independent samples for qPCR). **i** EnzS1-C4-2 cells were infected with pLKO and shARv7 viruses. And then the cells were treated w/o Enz for 2 and 6 days, the ARv7 and MAO-A levels were examined by qPCR; (*n* = 3 biological independent samples). **j** EnzS1 cells were infected by pWPI-ARv7, pLKO-shMAO-A, or both viruses. And then the cells were treated w/o 10 μM Enz and the cell viability analyzed by MTT assay; (*n* = 3 biological independent samples). For **c** and **f**–**i**, data represent the mean ± SEM, error bars represent SEM. *p*-value was determined by two-tailed paired *t*-test.

To dissect the molecular mechanism underlying Enz-induced MAO-A expression, we focused on the ARv7, as recent studies indicated the Enz-induced ARv7 is the key mechanism to induce the Enz resistance^14,28^. We first examined the effect of MAO-A inhibition in AR-negative PC3 cells, and found that adding clorgyline alone or clorgyline and Enz resulted in little effects on PC3 cell proliferation at 5 μM clorgyline (Fig. 3a) and 10 μM clorgyline (Fig. 3b), suggesting that Enz-increased MAO-A expression and effect of MAO-A inhibition is rather specific to AR-positive cells and may depend on AR (or ARv7) signals. As expected, adding Enz to EnzS1-C4-2 cells led to increase the expression of ARv7 and MAO-A at both mRNA and protein levels (Fig. 3c), In addition, adding ARv7-cDNA (OE-ARv7) also increased MAO-A mRNA expression in EnzR1-C4-2 cells (Fig. 3d, at mRNA level) and EnzS1-C4-2 cells (Fig. 3e, at both mRNA and protein levels), while suppressing ARv7 via adding ARv7-shRNA led to decrease MAO-A mRNA level in multiple EnzR cells, including EnzR1-C4-2, EnzR2-C4-2, and EnzR3-22Rv1 cells (Fig. 3f–h, respectively), as well as a decrease in MAO-A protein expression in the EnzR1-C4-2 and EnzR3-22Rv1 cells (Fig. 3f–h). To explore whether ARv7 induction by Enz is essential for the MAO-A increase, we treated EnzS1-C4-2-pLKO and EnzS1-C4-2-shARv7 cells w/o Enz, and then detected the ARv7 and MAO-A level. As shown in Fig. 3i, only in EnzS1-C4-2-pLKO cells, the ARv7 and MAO-A level can be induced by Enz treatment; however, the ARv7 and MAO-A expression cannot be induced by Enz in EnzS1-C4-2-shARv7 cells. To identify whether MAO-A is the key downstream gene of ARv7 to confer Enz resistance, we manipulated ARv7 and MAO-A expression in the EnzS1-C4-2 cells. As shown in the Fig. 3j, OE-ARv7 can decrease the Enz sensitivity; however, knockdown of MAO-A can reverse the ARv7 effects on Enz sensitivity, suggesting that ARv7 decrease Enz sensitivity dependent on MAO-A.

Finally, to test whether clorgyline can also suppress the expression of MAO-A (and ARv7), in addition to suppress the MAO-A activity, we found that adding clorgyline failed to impact significantly the expression of MAO-A (and ARv7) in the EnzR1-C4-2 cells during 3 days treatment (Supplementary Fig. 3b), suggesting that clorgyline’s effect on the altering Enz sensitivity could be mainly from suppressing the MAO-A activity.

Together, results from Fig. 3a–j suggest that Enz may function via increasing ARv7 to increase MAO-A expression during the development of Enz resistance.

### ARv7 transcriptionally increases the MAO-A expression

**Fig. 4:**
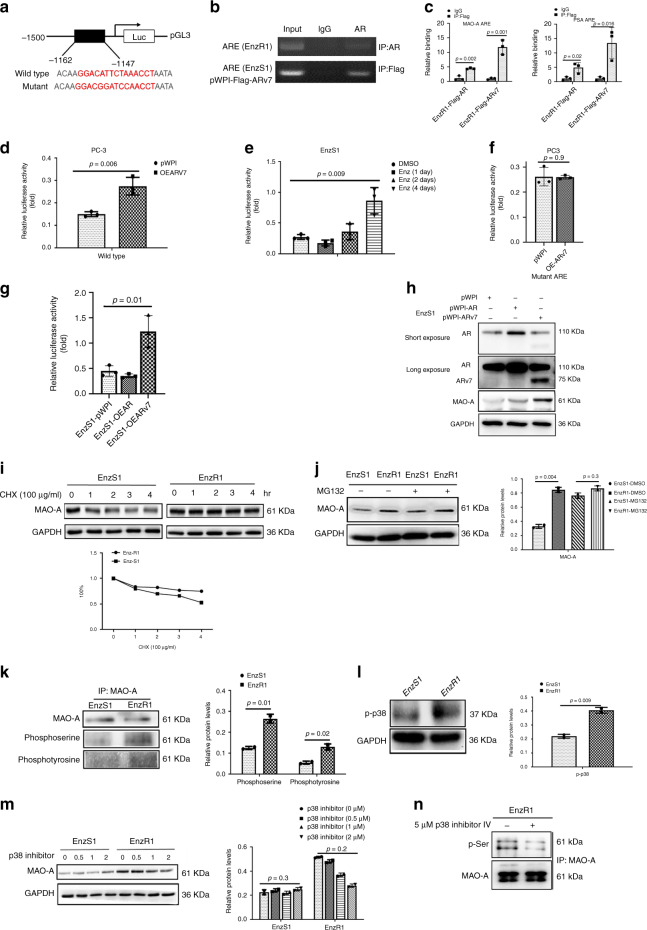
Enz upregulates the MAO-A transactivation in transcriptional level and protein level. **a** Schematic depiction of putative ARE on MAO-A promoter region. The mutant ARE was marked by italic font. **b** ChIP assay was performed to identify that endogenous AR and Flag-ARv7 bind to the putative ARE on MAO-A promoter in EnzR1-C4-2 and EnzS1-pWPI-flag-ARv7 cells. **c** EnzR1 cells were infected by Flag-ARv7 or Flag-ARfl virus. The ChIP assay was performed to analyze Flag-AR and Flag-ARv7 binding on MAO-A promoter region (left) and PSA promoter region (right) in EnzR1 cells; (*n* = 3 biological independent samples). **d** PC3 pWPI and pWPI-ARV7 cells were transfected by the PGL3-MAO-A promoter plasmid, and the promoter activity was determined by luciferase assay; (*n* = 3 biological independent samples). **e** EnzS1-C4-2 cells were treated with Enz at different time points and the MAO-A promoter activity was identified by luciferase reporter assay; (*n* = 3 biological independent samples). **f** MAO-A promoter with mutated ARE was constructed into PGL3 plasmid, and then the MAO-A promoter activity was analyzed in PC3 and PC3-oeARv7 cells; (*n* = 3 biological independent samples). **g** ARv7 and ARfl were transfected into EnzS1-C4-2 cells. And then the PGL3-MAO-A promoter luciferase activity was assayed; (*n* = 3 biological independent samples). **h** ARv7 and ARfl were transfected into EnzS1-C4-2 cells. And then the MAO-A expression was analyzed by western blot (WB). **i** EnzS1-C4-2 and EnzR1-C4-2 cells were treated with CHX for different time points. The MAO-A protein level was analyzed by WB. **j** EnzS1-C4-2 and EnzR1-C4-2 cells were treated with MG-132. The MAO-A protein level was analyzed by WB. **k** MAO-A phosphorylation is increased in EnzR1-C4-2 cells and the lysine phosphorylation of MAO-A was detected by specific antibody. **l** The phosphorylation of p38 (p-p38) protein levels were detected by WB in EnzS1-C4-2 and EnzR1-C4-2 cells. **m** EnzS1-C4-2 and EnzR1-C4-2 cells were treated with different concentrations of p38 inhibitor IV (0, 0.5, 1, and 2 μM) for 48 h. The MAO-A protein levels were analyzed by WB. **n** EnzR1-C4-2 cells were treated with p38 inhibitors for 48 h, and then the MAO-A protein was pulled down, MAO-A p-Ser level was examined by WB. For **c** and **d**, data represent the mean ± SEM, error bars represent SEM. *p*-value was determined by two-tailed paired *t*-test.

Next, to dissect the mechanism of how Enz-increased ARv7 can increase the MAO-A expression at the molecular level, we searched for the ARv7 response elements (AREs) on the 3 kb MAO-A promoter region, and found a putative ARE on the 5′ promoter region (Fig. 4a). We first checked ARv7 binding on this ARE and chromatin immunoprecipitation (ChIP) assay showed strong binding signal of Flag-ARv7, but not endogenous full-length AR (ARfl) (Fig. 4b). To better compare the binding ability of ARv7 and ARfl, we then overexpressed Flag-ARfl and Flag-ARv7 in EnzR1-C4-2 cells (which were maintained in 10 μM Enz) and used Flag antibody to pull-down ARfl and ARv7, suggesting that ARv7 has much stronger activity to bind in vivo to this ARE on the MAO-A promoter region as compared to ARfl (Fig. 4c). Such result is consistent with the previous reports that in EnzR cells, ARfl activity is suppressed significantly.

To confirm the results of ChIP assay, we checked AR and ARv7 binding on 5 kb MAO-A promoter region through ChIP-seq database (GEO: GSE106559). The results showed that in the presence of DHT, AR and ARv7 can bind to MAO-A promoter in different areas (Supplementary Fig. 4a). However, without DHT, AR lost its ability to bind to MAO-A promoter, yet ARv7 still showed very strong binding signal (Supplementary Fig. 4b). Interestingly, according to ChIP-seq data, although the strongest signal of ARv7 binding is on 4.5 kb upstream of MAO-A coding region, the ARv7 can still bind to the predicted ARE area in the absence of DHT. This data is consistent with our Fig. 4b, c, showing ARv7 has stronger activity to bind to MAO-A promoter.

Last, we also quantitated the enrichment of AR or ARv7 binding on MAO-A promoter region. As shown in Supplementary Fig. 4c, ARv7 binding in both conditions (w/o DHT) is stronger than AR.

Together, results from multiple approaches all confirmed that ARv7 has stronger binding ability than AR.

Results from luciferase assay via constructing the 3 Kb MAO-A promoter containing this ARE (or mutant ARE) into PGL3 reporter plasmid also confirmed that treating with Enz to increase ARv7 expression or direct adding ARv7-cDNA (OE-ARv7) could increase the MAO-A expression at the transcriptional level with wild-type ARE in both PC3 and EnzS1-C4-2 cells (Fig. 4d, e), and not with mutant ARE (see sequences in Fig. 4a) in the PC3 cells (Fig. 4f).

To further confirm ARv7, and not flAR, has better effects to increase MAO-A expression, we overexpressed ARfl and ARv7 in the EnzS1-C4-2 cells (cultured in 10% FBS RPMI that has 3 nM DHT), and then assayed the MAO-A promoter activity. As shown in Fig. 4g, only ARv7, and not ARfl, could enhance MAO-A promoter activity, suggesting that ARv7 had stronger ability to promote the MAO-A transcription.

To further confirm this data, we also assayed the MAO-A expression in EnzS1-AR and EnzS1-ARv7 cells. As shown in Fig. 4h, both ARfl and ARv7 can increase MAO-A expression; however, ARv7 showed much better effect compared to ARfl.

Together, results from multiple assays, including qPCR, western blot, ChIP-on in vivo binding assay, and luciferase reporter assay (Fig. 4a–h) all demonstrate that Enz-increased ARv7 expression may lead to increase MAO-A expression by transcriptional regulation via direct binding to the ARE on the MAO-A 5′ promoter region.

### ARv7 can increase the MAO-A protein stability

In addition to the transcriptional regulation, we also investigated whether Enz-increased ARv7 can lead to increase the MAO-A expression via protein stability. By treating EnzS1-C4-2 and EnzR1-C4-2 cells with the protein synthesis inhibitor cycloheximide (CHX), we found the MAO-A protein degradation rate is much lower in EnzR1-C4-2 cells than that in the EnzS1-C4-2 cells (Fig. 4i), suggesting that MAO-A protein is more stable in EnzR1-C4-2 cells than in EnzS1-C4-2 cells. In contrast, we found that the MAO-A protein expression in EnzS1-C4-2 and EnzR1-C4-2 cells was similar after adding the proteasome inhibitor, MG-132, suggesting that the degradation of MAO-A protein by the proteasome system is suppressed in EnzR1-C4-2 cells (Fig. 4j).

To further dissect the mechanism why the MAO-A protein stability is higher in EnzR1-C4-2 cells, we compared the phosphorylation of MAO-A in EnzS1-C4-2 and EnzR1-C4-2 cells, since early reports suggested that the phosphorylation could influence protein stability^30,31^. The results from immunoprecipitation of MAO-A from EnzS1-C4-2 and EnzR1-C4-2 cells revealed that serine phosphorylation (p-Ser) significantly increased in EnzR1-C4-2 cells (Fig. 4k).

We also assayed the p38 effects since it might also phosphorylate MAO-A to alter the MAO-A protein stability, and results revealed that the phosphorylation of p38 (p-p38) significantly increased in EnzR1-C4-2 cells (Fig. 4l), and adding the p38 inhibitor could only reduce MAO-A expression and p-MAO-A level in EnzR1-C4-2 cells but not in EnzS1-C4-2 cells (Fig. 4m, n), suggesting that p38 activity was enhanced in EnzR1-C4-2 cells and indicating that MAO-A was phosphorylated by higher p38 activity to increase the protein stability.

Taken together, results from Fig. 4i–n suggest that in addition to increase MAO-A expression via transcriptional regulation, Enz can also increase MAO-A expression via increasing its protein stability.

### Targeting the MAO-A suppressed hypoxia signals to overcome Enz resistance

**Fig. 5:**
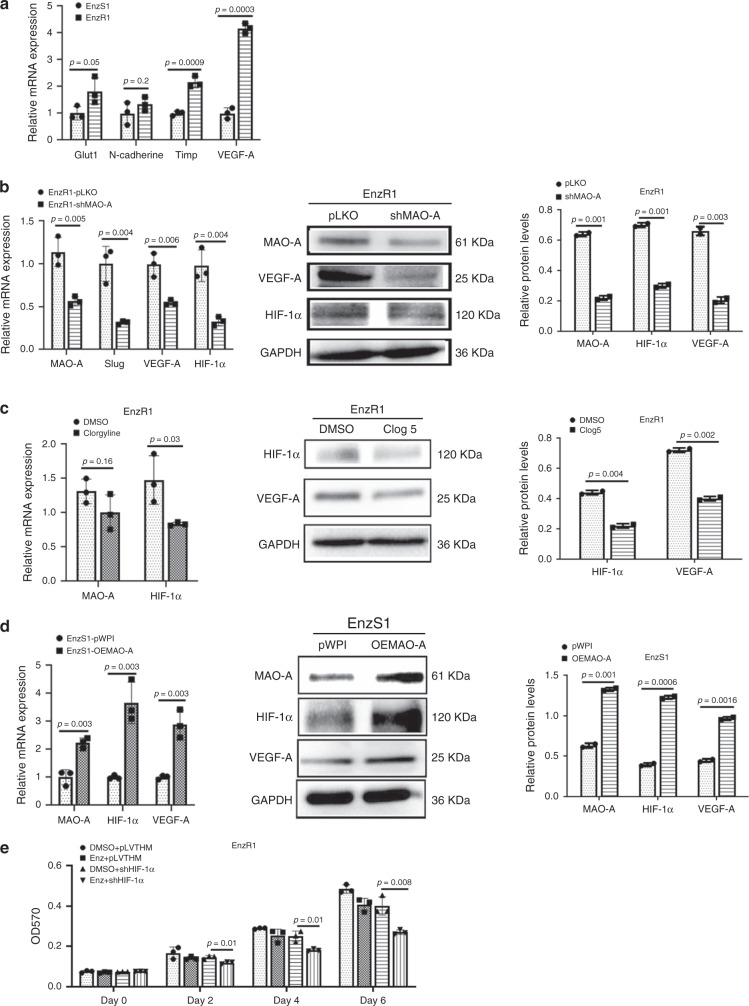
MAO-A activates hypoxia signaling to promote the Enz resistance. **a** The mRNA levels of Glut1, N-Cadherin, Timp, and VEGF-A in EnzS1-C4-2 and EnzR1-C4-2 cells were analyzed by qPCR; (*n* = 3 biological independent samples). **b** In EnzR1-C4-2 cells, MAO-A was knocked down by shRNA. And the mRNA levels of MAO-A, Slug, VEGF-A, and HIF-1α were analyzed by qPCR (left), as well as HIF-1α and VEGF-A expression by western blot (right); (*n* = 3 biological independent samples for qPCR). **c** EnzR1-C4-2 cells were treated with/without 5 μM clorgyline, and the mRNA and protein level of MAO-A, HIF-1α, and VEGF-A were analyzed by qPCR (HIF-1α and MAO-A, left) and western blot (HIF-1α and VEGF-A, right); (*n* = 3 biological independent samples for qPCR). **d** MAO-A was overexpressed (oeMAO-A) in EnzS1-C4-2 cells, and the mRNA and protein levels of MAO-A, HIF-1α, and VEGF-A were analyzed by qPCR (left) and western blot (right); (*n* = 3 biological independent samples for qPCR). **e** HIF-1α was knocked down (shHIF-1α) in EnzR1-C4-2 cells, and then the cells were treated with/without Enz. The cell viability was analyzed by MTT assay; (*n* = 3 biological independent samples). Data represent the mean ± SEM, error bars represent SEM. *p*-value was determined by two-tailed paired *t*-test.

Next, to dissect the mechanisms of how targeting the Enz/ARv7/MAO-A signaling can overcome the Enz resistance in multiple EnzR cells, we focused on hypoxia signals since recent studies indicated MAO-A might exert its biological functions via altering the key hypoxia signals^24^. We first examined the MAO-A effects on the hypoxia signals with the HIF-1α and its downstream target genes, including the Glut1, N-Cadherin, Timp, and VEGF-A. The results revealed higher expression of these hypoxia downstream genes in EnzR1-C4-2 cells than those in EnzS1-C4-2 cells (Fig. 5a). Suppressing MAO-A with MAO-A-shRNA (Supplementary Fig. 5a) also led to reduce the expression of HIF-1α and its target genes expression at mRNA (Fig. 5b, left) and the protein levels of HIF-1α and VEGF-A (Fig. 5b, right) in EnzR1-C4-2 cells. Suppressing HIF-1α with HIF-1α-shRNA also reduced its target gene VEGF-A expression at mRNA and protein levels in EnzR1-C4-2 cells (Supplementary Fig. 5b).

In contrast, treating with MAO-A inhibitor clorgyline decreased these hypoxia-related genes at mRNA and protein levels in EnzR1-C4-2 cells (Fig. 5c).

Using an opposite approach via increasing MAO-A via adding MAO-A-cDNA in EnzS1-C4-2 cells also resulted in increasing the target genes HIF-1α and VEGF-A expression at both mRNA and protein levels (Fig. 5d). More importantly, our results showed that knocking down HIF-1α led to increase the Enz sensitivity in EnzR1-C4-2 cells (Fig. 5e), which suggested that MAO-A can promote EnzR via activating HIF-1α signaling.

Together, results from Fig. 5a–e suggest that Enz/ARv7/MAO-A axis can overcome the Enz resistance via altering the hypoxia signals in EnzR1-C4-2 and EnzS1-C4-2 cells.

### Targeting MAO-A signaling suppressed EnzR tumor growth in vivo

**Fig. 6:**
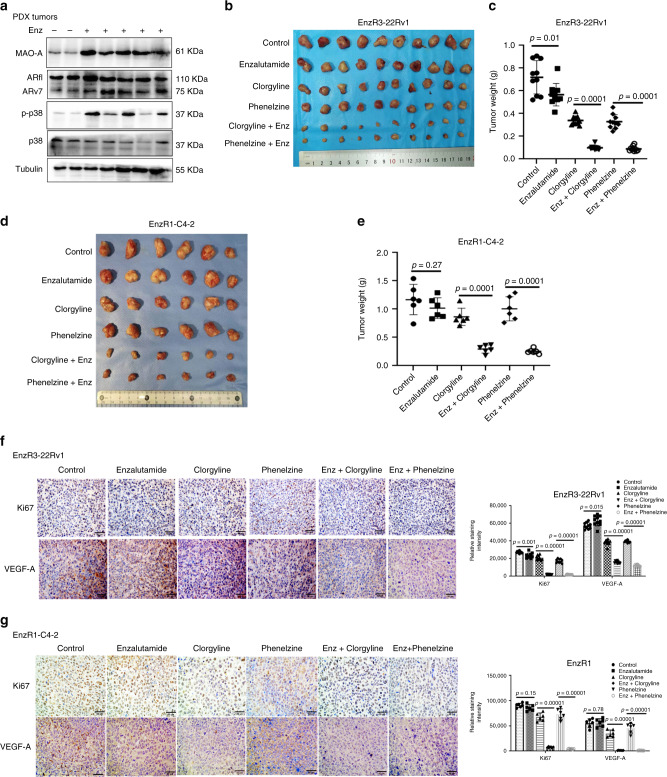
Clorgyline and phenelzine, inhibitors of MAO-A, can overcome Enz resistance. **a** The in vivo PDX-PCa mouse model data revealed that injection with Enz (30 mg/kg/every other day) increased the MAO-A, ARv7, and p-p38 level. **b**, **c** Mice (*n* = 10) implanted with EnzR3-22Rv1 xenografts were treated with vehicle control, Enz (30 mg/kg), clorgyline (10 mg/kg), phenelzine (30 mg/kg), Enz+clorgyline (30 mg/kg + 10 mg/kg), or Enz+phenelzine (30 mg/kg + 30 mg/kg). After sacrifice, tumors of the six groups were collected and weighed. **d**, **e** Mice (*n* = 6) implanted with EnzR1-C4-2 xenografts received the same treatments as EnzR3-22Rv1. After sacrifice, tumors of the six groups were collected and weighed. **f** IHC staining of Ki67 and VEGF-A in EnzR3-22Rv1 tumors were performed; (scale bar = 50 μm). **g** IHC staining of Ki67 and VEGF-A in EnzR1-C4-2 tumors were performed; (scale bar = 50 μm). Data represent the mean ± SEM, error bars represent SEM. *p*-value was determined by two-tailed paired *t*-test.

To establish a preclinical proof-of-principle in the in vivo mouse model, we first established the in vivo PDX-PCa mouse model in six mice, and results revealed that mice receiving Enz (30 mg/kg/every other day) had an increase in MAO-A, ARv7, and p-p38 level in the xenografted PDX tumors (Fig. 6a).

We then established the second in vivo mouse model by implanting EnzR3-22Rv1 cells in the nude mice for the purpose of examining the effects of clorgyline and phenelzine on EnzR tumors. To make sure all data are repeatable and reach the statistical significance, we performed two sets of experiments, with implanted EnzR3-22Rv1-Luc cells (cells which express luciferase plasmid) in the first set and implanted EnzR3-22Rv1 cells in the second set into nude mice. Once the tumor formation was detectable (after 4 weeks), the mice were treated in groups as follows: (1) vehicle, (2) Enz (30 mg/kg), (3) clorgyline (10 mg/kg), (4) phenelzine (30 mg/kg), (5) Enz+clorgyline, and (6) Enz+phenelzine, and i.p. injected every 2 days for 4 weeks. For EnzR3-22Rv1-Luc cells, we used in vivo imaging system (IVIS) to monitor the tumor sizes weekly. After 4 weeks injections, we sacrificed the mice (four mice/group in the first set and ten mice/group in the second set) 2 days after the final treatment, and then measured the tumor weights.

The results revealed that adding Enz alone has little effect on the EnzR3-22Rv1 (Fig. 6b) or EnzR3-22Rv1-Luc (Supplementary Fig. 6a, b) growth. In contrast, combining Enz with clorgyline or phenelzine significantly suppressed EnzR3-22Rv1 (Fig. 6b) or EnzR3-22Rv1-Luc (Supplementary Fig. 6a, b) tumors growth. Tumor volumes and weights after sacrifice also confirmed that combining Enz and clorgyline or phenelzine can suppress EnzR3-22Rv1 and EnzR3-22Rv1-Luc tumors progression in mice compared with Enz alone and control vehicle group (Fig. 6c, Supplementary Fig. 6c).

Finally, to further confirm the above two in vivo mouse models (PDX and EnzR3-22Rv1), we also established the third in vivo mouse model with implanting another EnzR (EnzR1-C4-2) cells into B-NDG mice and repeated the treatments on EnzR3-22Rv1 tumors. As expected, we obtained the similar results showing clorgyline and phenelzine can also reverse the EnzR1-C4-2 resistance. (Fig. 6d, e)

We also performed the IHC staining on EnzR3-22Rv1, EnzR3-22Rv1-Luc, and EnzR1-C4-2 tumors to assay the proliferation markers Ki67 and VEGF-A, and results showed that in all three EnzR tumors, the expression of these proliferation proteins were significantly inhibited by the combination treatments (Fig. 6f, g, Supplementary Fig. 6d).

Together, results from Fig. 6a–g and Supplementary Fig. 6a–d using three preclinical in vivo mouse models all conclude that combining Enz with the MAO-A inhibitors clorgyline or phenelzine can overcome Enz resistance to further suppress the growth of EnzR tumors in the well-established EnzR in vivo models.

## Discussion

Therapeutic regimens targeting the AR are effective for patients with advanced PCa, including those who failed first-line traditional ADT. A variety of mechanisms underlying disease progression on these regimens involve aberrant reactivation of AR signaling^32^. The detailed mechanisms for the development of antiandrogen resistance, especially to the potent antiandrogen Enz, however, remain unclear. Korpal et al. found that AR-F876L mutation might confer resistance to Enz^6^. Liu et al. also found that the AKR1C3 might enhance the Enz resistance via increasing the cellular androgen level^33^, and GR might also contribute to the Enz resistance via replacing AR to activate the cellular survival pathways^8^. However, among many potential mechanisms leading to Enz resistance, the Enz-induced AR splice variant ARv7 received the most attention due to strong clinical data support. Importantly, ARv7 can be measured noninvasively in patients with mCRPC via their blood samples CTCs, and positive detection of ARv7 has been consistently linked to poor prognosis and poor response to Enz. Currently, there are no effective therapies for patients with positive detection of ARv7, highlighting the priority in developing novel agents to overcome the lethal EnzR CRPC^14^.

Several previous approaches targeting ARv7 in CRPC have been developed in preclinical studies. These approaches include therapeutic targeting of the AR with the AR degradation enhancer ASC-J9^34,35^, Niclosamide^36^, and Galeterone (TOK-001)^37^. However, most of these inhibitors are in the different stages of clinical trials, and the Galeterone trial has failed. Direct targeting of ARv7, which is a transcription factor, can be challenging. In addition, clinical development of novel agents is time consuming, even given the promising initial clinical results, mainly due to many regulatory hurdles that new drugs need to overcome before approved for use in humans. Repurposing existing drugs represents an effective approach to address these limitations.

As a key enzyme to control the production of neurotransmitters, MAO-A also has been demonstrated to play the important roles in PCa. Recent studies demonstrated that MAO-A could promote the PCa metastases to bone via activating the shh-Rankl signal and cancer stem cells^21,38^. Other study suggested that targeting MAO-A with clorgyline in PCa VCaP cell could also strongly suppress the cell proliferation^23^. These studies provide the solid evidence that targeting the MAO-A in advanced PCa might represent a potential therapeutic strategy to suppress PCa progression. However, there is no study suggesting that MAO-A can be also used to treat the CRPC that already developed Enz resistance.

In this study, we show that MAO-A is a valid target to overcome Enz resistance in CRPC. First, we discovered MAO-A is activated by Enz treatment. Second, higher MAO-A expression is associated with, and regulated, by ARv7. Third, MAO-A may function via altering the downstream hypoxia/HIF-1α signaling to increase the Enz resistance.

An early study indicated that dopamine could also function as MAO-A substrate to alter its activity, and another study demonstrated that dopamine D2 receptor agonist could enhance the PCa chemotherapy efficacy, suggesting that dopamine may suppress the PCa cells growth^39^. Interestingly, another study also showed that dopamine D2 receptor is decrease in hypoxia, which provide the evidence of the negative correlation between dopamine and HIF-1α, however, the positive correlation between MAO-A and HIF-1α^40^.

Most importantly, we demonstrate here that antidepression drugs, including clorgyline and phenelzine, are effective in suppressing EnzR CRPC cell growth and can restore Enz sensitivity in preclinical models. Instead of direct targeting the ARv7, clorgyline and phenelzine target the ARv7 key downstream gene MAO-A to alter the ARv7-induced Enz resistance. The advantage of using clorgyline or phenelzine includes to overcome Enz resistance in the EnzR cells (Fig. 2b–d), and to delay the development of Enz resistance when combined with Enz in the EnzS cells (Fig. 2j) with few side effects, as phenelzine has been used to treat the depression, (and sometimes for Parkinson’s disease) for a long time^19^. With established toxicity profiles of this drug in humans, they may be able to be applied to the treatment of EnzR CRPC patients on an accelerated path, either as a single agent or in combination with Enz for the purpose of evaluating their potential to overcome tumor resistance^41,42^.

The monoamine oxidase inhibitors (MAOIs) were originally synthesized in the 1950s^43^. Although initially developed to modulate neuron diseases by altering the levels of MAO substrates, the use of MAOIs is now an area of great interest^44^. The dosage of clorgyline in human patients with neuron diseases is 30–40 mg/kg^27^, and in the mouse models for neuron diseases or PCa is 10 mg/kg/24 h^24,45^, and in the in vitro cell lines are 10 μM in neuron disease (glioma)^45^. For phenelzine used as an antidepressant, the dose is 45–60 mg/kg^46^ in humans, 30 mg/kg^47^ in mouse models, and 40–50 μM^48,49^ was used in in vitro neuron or PCa cell lines. For both drugs, our doses used were 5–10 μM in the in vitro cell lines and 10–30 mg/kg every other day in the in vivo mouse model, which were lower than the FDA-approved human usage doses, further supporting the feasibility of administrating safe doses of these drugs for the purpose of overcoming the Enz resistance in the CRPC.

In our study, we analyzed MAO-A expression in CTCs samples from patients who received Enz treatment. We acknowledge that increased MAO-A gene expression detected in post-Enz CTC sample may reflect, at least in part, the increased CTC number in such samples. We did, however, show that while MAO-A is significantly higher in post-Enz CTC samples in those who turned ARv7 positive, the same was not observed for CTC ARfl data (Supplementary Fig. 2). Our approach is limited by the fact that many CTC-specific control genes can not serve as reliable and validated control genes. We inquired about the possibility of using a relatively stable prostate specific marker, such as HOXb13 as the control gene. However, this was a retrospective study using “left-over” cDNA. The cDNA for this set of samples has been depleted and not available for additional analysis.

In Fig. 6a, we confirmed that adding Enz led to increase ARv7 and MAO-A expression in PDX samples. However, we also found that the increase in MAO-A after Enz treatment in this PDX sample did not match perfectly with increased ARv7, suggesting other factor(s) may also contribute the increased MAO-A expression. Indeed, results from Fig. 4 also indicated that p38 could also play positive roles to stabilize MAO-A protein (and therefore increasing MAO-A protein expression), and data in the Fig. 6a also showed that Enz could increase the p38 activity (p-p38) in PDX tumors, suggesting that both ARv7 and p38 may coordinate to upregulate the MAO-A expression in CRPC cells after Enz treatment.

In summary, our study demonstrates clorgyline or phenelzine, the existing antidepression MAO-A inhibitors can overcome Enz resistance and further suppress EnzR cell growth. Repurposing these drugs as a new therapy, either a single agent or in combination with the current standard Enz treatment, may overcome Enz resistance and further extend the survival benefit to men with EnzR CRPC.

## Methods

### Cell culture and reagents

CWR22Rv1 VCaP and PC3 cell lines were purchased from the American Type Culture Collection (ATCC, Manassas, VA) and cultured in RPMI 1640 with 10% FBS. HEK293T cells were purchased from the ATCC (Manassas, VA) and cultured in DMEM with 10% FBS. The EnzS1-C4-2 cell line was a gift from Dr. Leland W. K. Chung from Cedars-Sinai. EnzS4-C4-2B and EnzR4-C4-2B cell lines were gifts from Dr. Allen Gao from UC Davis. C4-2 EnzR cell lines were generated via chronic culture of CRPC C4-2 cells in media containing increasing Enz (from 10 μM to 30 μM), with the increased concentration added when cells were no longer sensitive (EnzR1-C4-2) or continuous culture with 10 μM Enz for 6 months (EnzR2-C4-2). All cells were maintained in a humidified 5% CO_2_ environment at 37 °C. All cell lines have been authenticated by ATCC and periodically reauthenticated by PCR, and determined to be mycoplasma and bacteria free following ATCC’s instructions. The CHX, p38 map kinase inhibitor IV, and clorgyine were purchased from Sigma-Aldrich (St. Louis, MO). Enz was purchased from MedChemExpress (South Brunswick, New Jersey).

### Cell proliferation assays

We plated 1 × 10^4^ C4-2, PC3, and C4-2 EnzR cells into each well of 24-well plates for MTT assays on days 0, 2, 4, and 6 for the PCa cells and on days 0, 1, 2, and 3 for the EnzS1-C4-2 and EnzR1-C4-2. After the various time points, the MTT assay was performed by adding 100 μl of 5 mg/ml MTT to each well. We included one set of wells with MTT, but no cells (control), then incubated for 3 h at 37 °C, removed media and added 150 μl DMSO, covered the plates with foil, agitated the cells on an orbital shaker for 15 min, and then read the absorbance at 570 nm.

### Lentivirus packaging and cell transfection

The shARv7 was constructed into the pLKO.1 lentiviral vector as reported previously. The pLKO.1 shMAO-A, together with package and envelope plasmids, psPAX2 and pMD2G, were cotransfected into 293 T cells for 48 h to produce the MAO-A shRNA lentivirus particle soup, which was then collected and frozen at −80 °C for later use in transduction of PCa cells.

### RNA extraction and quantitative real-time PCR

Total RNAs were isolated using Trizol reagent (Invitrogen, Grand Island, NY). One μg of total RNA was subjected to reverse transcription using Superscript III transcriptase (Invitrogen). Real-time PCR (RT-PCR) was conducted using a Bio-rad CFX manager 3.0 system with SYBR green to determine the mRNA expression level of a gene of interest. Expression levels were normalized to GAPDH level.

### RNAseq

The RNAseq of EnzS1-C4-2 and EnzR1-C4-2 cells were done by UR genomic research center. The mRNA was treated with DNase I and was fragmented for cDNA library construction. Then, the cDNA was synthesized with random hexamer primers and was further subjected to end repair and adapter ligation using T4 DNA ligase. The products of ligation reaction were purified and cDNA fragments (~200 bp) were recovered. PCR was carried out to enrich the purified cDNA template. After validating on Quit and Bioanalyzer, the library was sequenced using Illumina HiSeq 2500 according to the manufacturer’s instruction.

### Western blot analysis

Cells were lysed in RIPA buffer and proteins were separated on 10% SDS/PAGE gel and then transferred onto PVDF membranes (Millipore, Billerica, MA). After blocking with 5% BSA-PBST buffer, the membranes were blotted by specific primary antibodies (1:1000 dilution) and HRP-conjugated secondary antibodies (1:5000 dilution). Finally, the signals on the membranes were visualized by ECL system (Thermo Fisher Scientific, Rochester, NY). We used the Bio-rad Image-lab 4.0.1 for collecting WB data. The MAO-A (sc-271123), GAPDH (sc-47724), tubulin (sc-23948), VEGF-A (sc-7269), AR (sc-816), p38 (sc-81621), and HIF-1α (sc-13515) antibodies were from Santa Cruz Biotechnology, Inc (Santa Cruz, CA). The p-p38 antibody (#9211) was purchased from Cell signaling Technology (Danvers, MA). Rabbit HRP-conjugated second antibody (G21234) and mouse HRP-conjugated second antibody (G21040) were from Invitrogen (Carlsbad, CA).

### MAO-A activity assay

MAO-Glo assay systems were purchased from Promega (Madison, WI). The cells were lysed by luciferase lysis buffer, and the cell lysates were applied to analyze the MAO-A activity by the MAO-Glo kit.

### Chromatin immunoprecipitation assay

A total of 10^7^ cells were cross-linked with 4% formaldehyde for 10 min and then incubated with 125 mM glycine 5 mins to quench the formaldehyde. The cells were sonicated to yield genomic DNA fragments of 300–1000 bp long. Lysates were precleared sequentially with normal rabbit IgG (sc-2027, Santa Cruz Biotechnology) and protein A-agarose in 4 °C for 1 h. Anti-Flag (MAB3118, Sigma), AR (sc-816, Santa Cruz) antibodies (2.0 µg) were added to the lysates and incubated at 4 °C overnight. For the negative control, IgG was used to incubate the cell lysates. Specific primers for the protein-binding DNA regions were designed to amplify a target sequences within and PCR products were analyzed by agarose gel electrophoresis or quantitative RT-PCR (qRT-PCR).

### Luciferase assay

PC3-Pwpi and PC3-oeARv7 cells were plated in 24-well plates and cotransfected with PGL3-MAO-A-promoter containing the WT-ARE or mutant ARE and pRL-TK, which is used as internal control using Lipofectamine (Invitrogen). After 48 h transfection, Luciferase activity was measured by Dual-Luciferase assay (Promega, Madison, WI) according to the manufacturer’s manual. The EnzS1 cells were transfected with PGL3-MAO-A-promoter containing the WT-ARE and pRL-TK using Lipofectamine. After 12 h of transfection, the cells were treated with 10 µM Enz for 1, 2, and 4 days, and then the luciferase activity was measured.

### PDX implantation and different compound treatments

The PCa-133 PDX samples are the gifts from Dr. Sankar N. Maity at MD Andersen Cancer Center^50^. PCa-133 tumors were chopped to small pieces (1 mm^3^), and implanted into six SCID mice subcutaneously^51^. After average tumor’s volumes reached 200 mm^3^, we i.p injected two mice with DMSO and five mice with Enz (30 mg/kg) every other day. After ten injections, the mice were sacrificed and the tumors were collected for WB. All the mice were purchased from NCI. All experiments were conducted after approval from University of Rochester medical center and follow the regulations of University Committee on Animal Resources (UCAR).

### In vivo tumorigenesis assay

EnzR3-22Rv1, EnzR3-22Rv1-luc, and EnzR1-C4-2 (1 × 10^6^) were mixed with Matrigel (1:1) and injected into the prostates of 6- to 8-week-old male nude mice (EnzR3-22Rv1-luc and EnzR3-22Rv1) or B-NDG mice (EnzR1-C4-2). Tumor-bearing mice were randomized into four groups and treated by i.p. every other day for 4 weeks as follows: (1) vehicle, (2) Enz (30 mg/kg), (3) clorgyline (10 mg/kg), (4) phenelzine (30 mg/kg), (5) Enz+clorgyline, and (6) Enz+phenelzine. For EnzR3-22Rv1-luc tumors, IVIS was used weekly to monitor tumor growth. We imaged the mice a final time 2 days after the final treatment, sacrificed the mice, and monitored tumor growth with the Perkin elmer IVIS for 22Rv1-luc tumors (EnzR3-22Rv1-luc), as well as tumor sizes and tumor weights. All experiments were conducted after approval from University of Rochester Medical Center and Harbin Medical University and follow the regulations of UCAR.

### Analysis of CTC

CTC samples used for MAO-A expression analysis were excess “left-over” cDNA samples from an ongoing prospective blood-based CTC ARv7 study in men with metastatic CRPC. Samples were from patients enrolled in a prospective biomarker study. Patients were treated with standard-of-care therapies (abiraterone, Enz, and docetaxel), and biomarker results were not utilized to make treatment decision. These cDNA samples were prepared using the commercially available Adnatest platform (Qiagen, Hanover, Germany) designed for blood processing, CTC isolation, and cDNA preparation^29^. This study was approved by the Johns Hopkins University institutional review board, and patients provided written informed consent. Selection for left-over CTC samples for MAO-A expression analysis involved a retrospective review of available cDNA samples prepared from CTCs and stored at −80 °C. A total of 288 frozen cDNA samples were selected on the basis of availability of adequate cDNA for analysis, and processed for quantitative PCR analysis on CFX96 Touch Real-Time PCR Detection System (Bio-Rad, Hercules, CA). PCR cycles at 95 °C × 5 min, 40 cycles of 95 °C × 10 s, 58 °C × 30 s, and 72 °C × 30 s were followed by melting curve analysis. Primer sequences used for MAO-A were 5′-AATTCAGCGGCTTCCAATGG-3′(forward) and 5′-CAAGTCGATCAGCTTTCCGG-3′(reverse); primer sequences used for RPL13A were 5′-CCTGGAGGAGAAGAGGAAAGAGA-3′ (forward) and 5′-TTGAGGACCTCTGTGTATTTGTCAA-3′ (reverse). Patient treatment status and sample collection time points were unblinded after laboratory data was generated. Among the 288 cDNA samples, 90 were CTC negative (CTC−), 127 were CTC positive (CTC+) but ARv7 negative (ARv7−), and 71 were CTC+ and ARv7 positive (ARv7+). MAO-A expression data were normalized to the control gene (RPL13A), and normalized data presented for each biomaker group according to CTC and ARv7 status, as well as each pretreatment and posttreatment pairs. Difference of MAO-A copy numbers according to CTC and ARv7 status were compared using two-tailed unpaired *t*-test. Changes of MAO-A expression before and after Enz treatment were compared by two-tailed paired *t*-test. *p*-values of 0.05 or less were considered statistically significant. All statistical tests were performed by GraphPad Prism version 7.02 (GraphPad software, San Diego, CA).

### Statistics and reproducibility

All experiments were performed in triplicate and at least three times. The data values were presented as the mean ± SEM. Differences in mean values between two groups were analyzed by two-tailed Student’s *t*-test and ANOVA. *p* ≤ 0.05 was considered statistically significant.

### Reporting summary

Further information on research design is available in the Nature Research Reporting Summary linked to this article.

## Supplementary information

Supplementary Information

Reporting Summary

## Data Availability

The data that support the findings of this study are within the Article, Supplementary Information, or available from the corresponding author upon reasonable request.
