# Supplementary material for: The MAO inhibitors phenelzine and clorgyline revert enzalutamide resistance in castration resistant prostate cancer
Source: Nat Commun. 2020 Jun 1;11:2689. doi: 10.1038/s41467-020-15396-5 (PMC7264333; doi:10.1038/s41467-020-15396-5)
Supplement: Supplementary file 1 — Supplementary Information [file 41467_2020_15396_MOESM1_ESM.docx]

**Supplementary information**

**
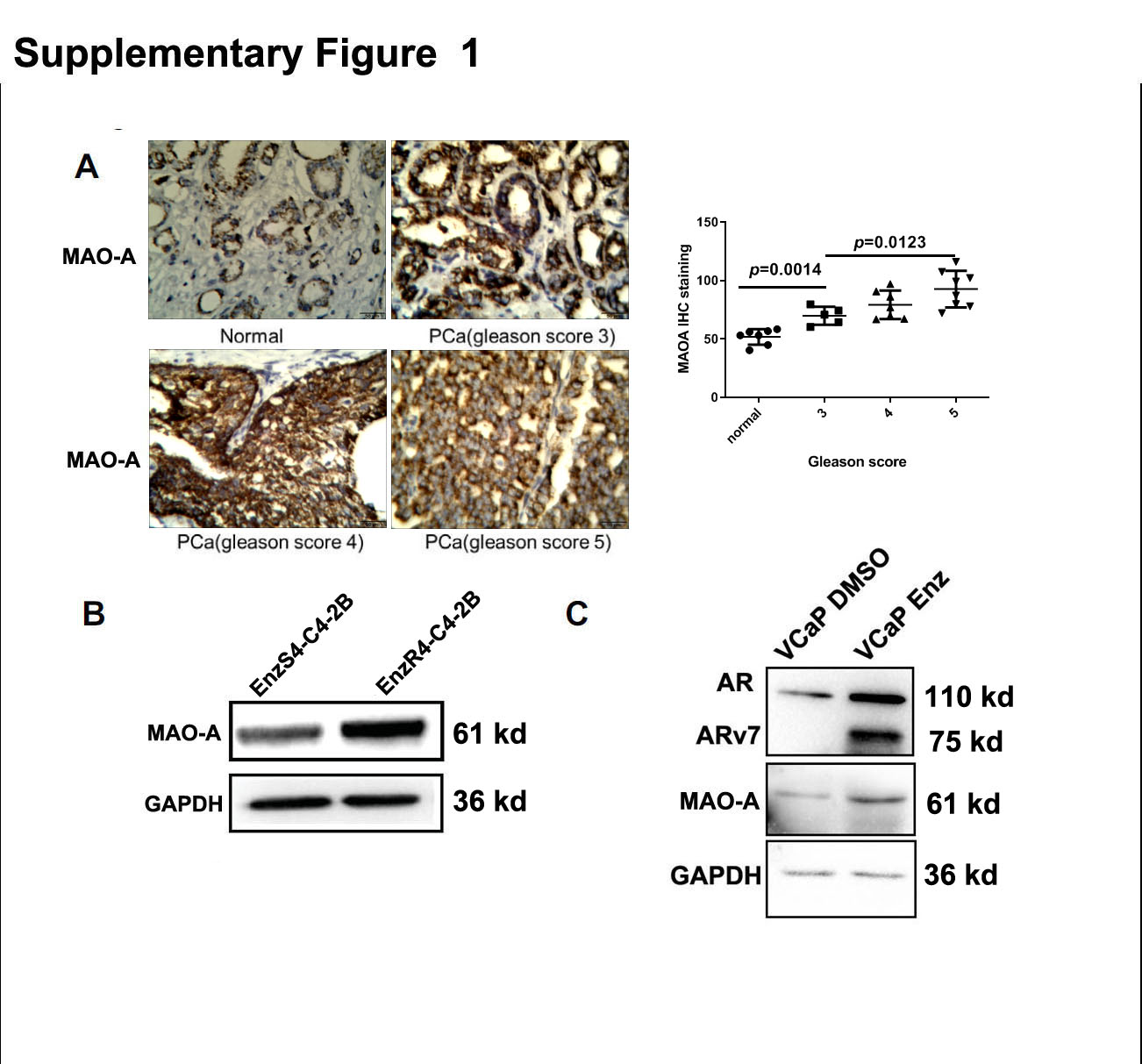
**

**Supplementary Figure 1**

(A) Quantitative IHC analysis of MAO-A protein expression in normal prostate (n = 7), PCa clinical samples (Gleason score 3) (n = 5), PCa clinical samples (Gleason score 4) (n = 7), and PCa clinical samples (Gleason score 5) (n = 8). Arrows indicate tumor areas. (B) Western blot analysis of MAO-A expression in EnzS4-C4-2B and Enz-R4-C4-2B cells. (C) VCaP cells were treated by Enz for 10 days and then the ARv7 and MAO-A protein levels were examined by WB. For A Quantitations are mean±SEM, P-value was determined by two-tailed paired t test.

**
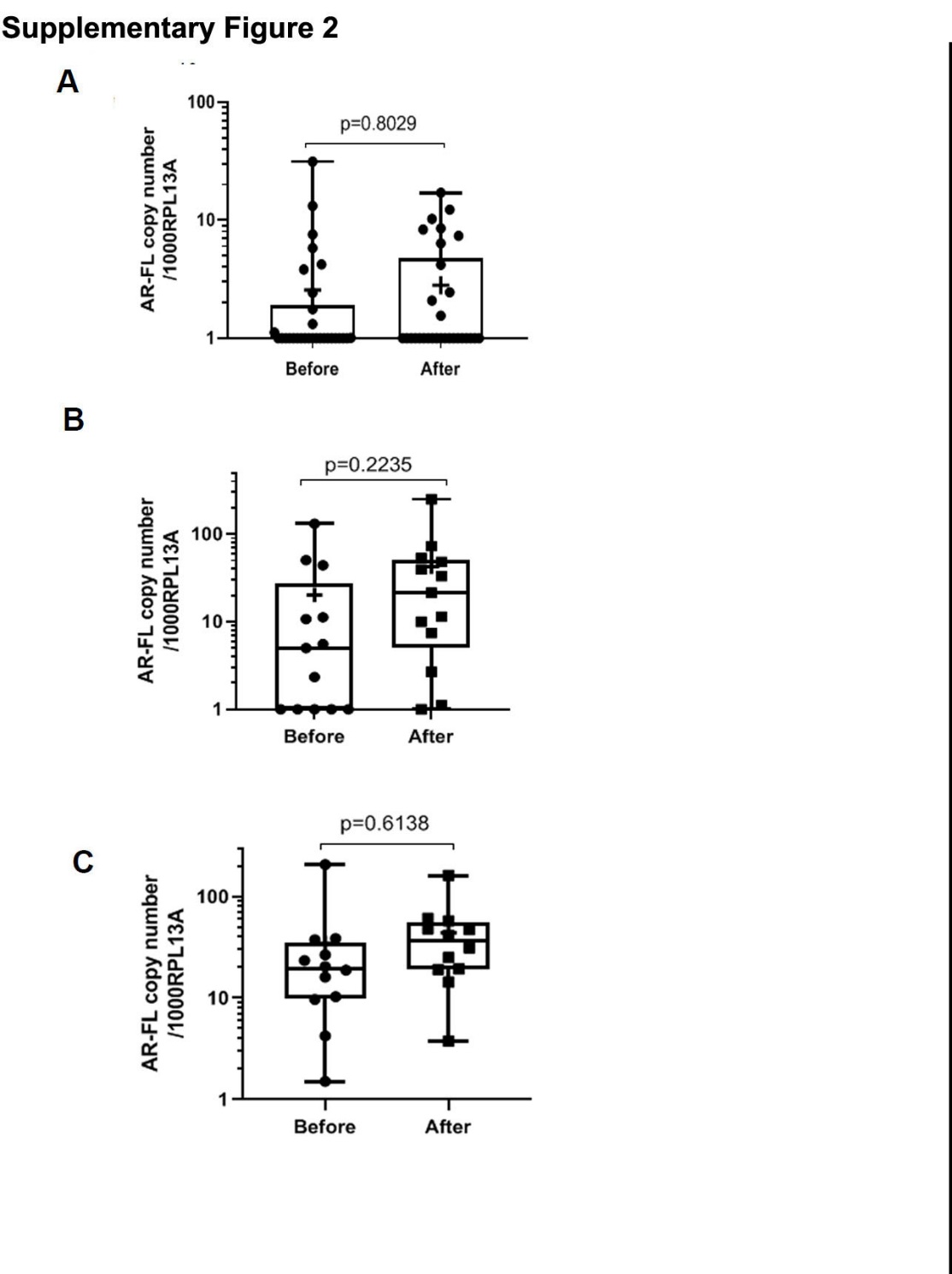
**

**Supplementary Figure 2**

ARfl copy numbers at baseline (before) and after Enzalutamide treatment (after) in patients whose ARv7 status (A) remained negative (N=30), (B) changed from negative to positive (N=13), and (C) remained positive (N=12) after Enzalutamide treatment. P-values were determined by two-tailed paired t-test.

**
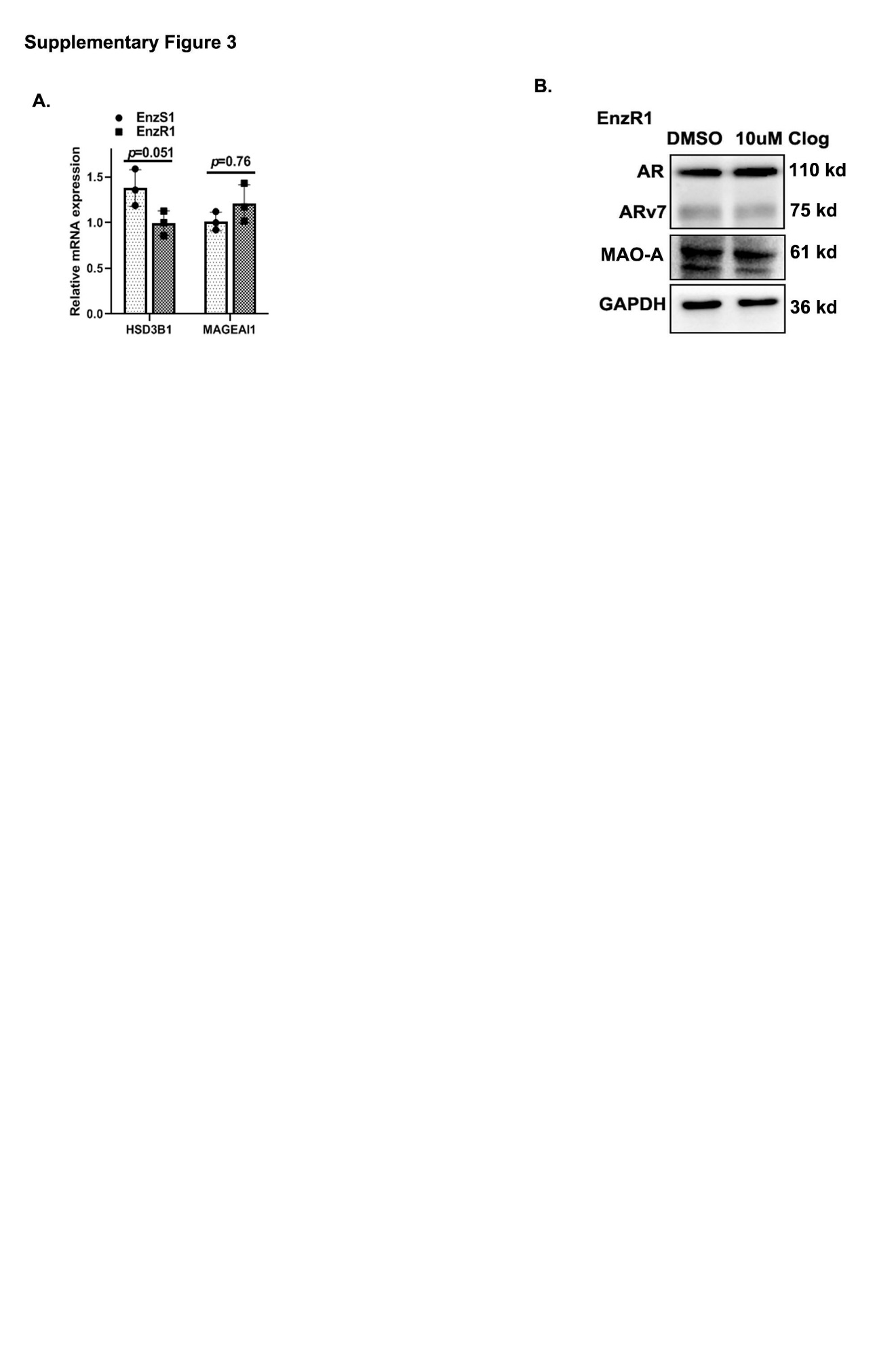
**

**Supplementary Figure 3**

(A) MGEA11 and HSD3B1 expression EnzR1 and EnzS1 cells were analyzed by qPCR. (B) The EnzR1 cells were treated with/without 10uM clorgyline (Clog) for 24 hours. The MAO-A and ARv7 expression were analyzed by WB. Quantitations are mean±SEM, P-value was determined by two-tailed paired t test.

**
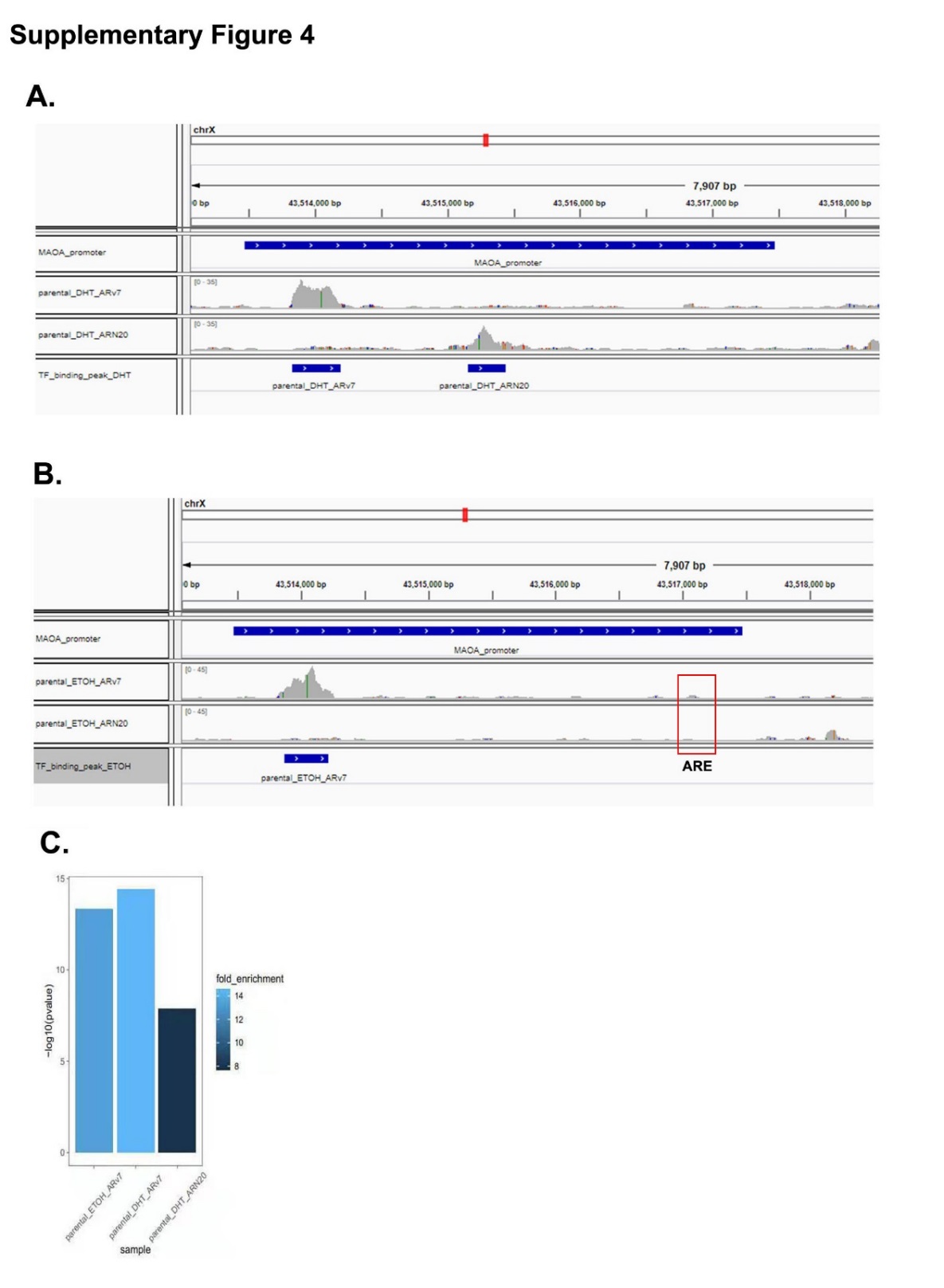
**

**Supplementary Figure 4** The ARN20 (ARfl) and ARv7 binding on MAO-A promoter regions in presence of DHT (A) or in the absence of DHT (ETOH) (B) were analyzed based on ChIP-seq data (GEO: GSE106559). (C) The quantification of ARN20 (ARfl) and ARv7 binding on the MAO-A promoter region.


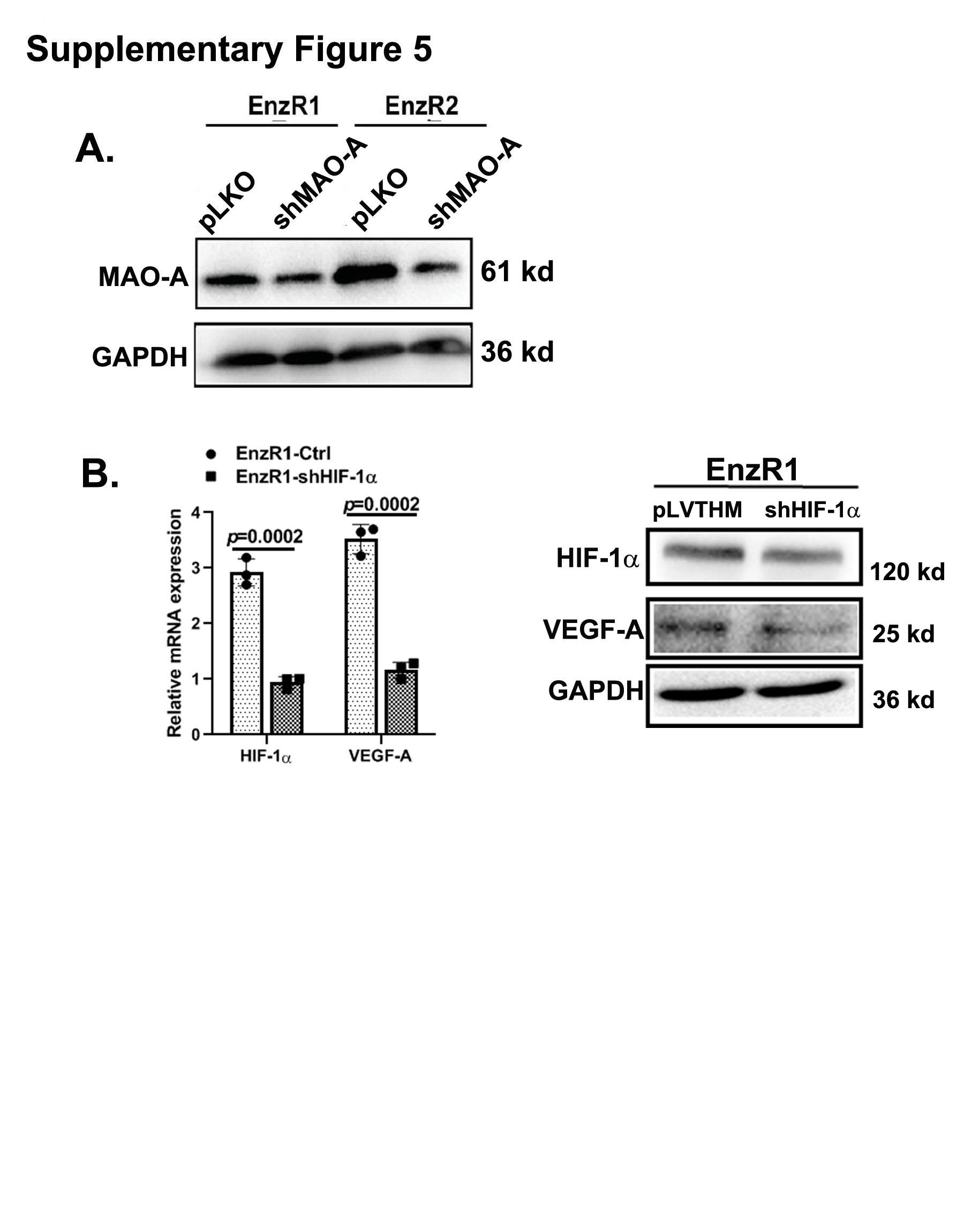


**Supplementary Figure 5**

(A) Western blot analysis to identify the knockdown efficiency of MAO-A in EnzR1-C4-2 and EnzR2 cells. (B) The qPCR analysis (left) to identify the mRNA level and Western blot analysis (right) of HIF-1α and VEGF-A in EnzR1-C4-2 pLVTHM and shHIF-1α cells. For B Quantitations are mean±SEM, P-value was determined by two-tailed paired t test.

**
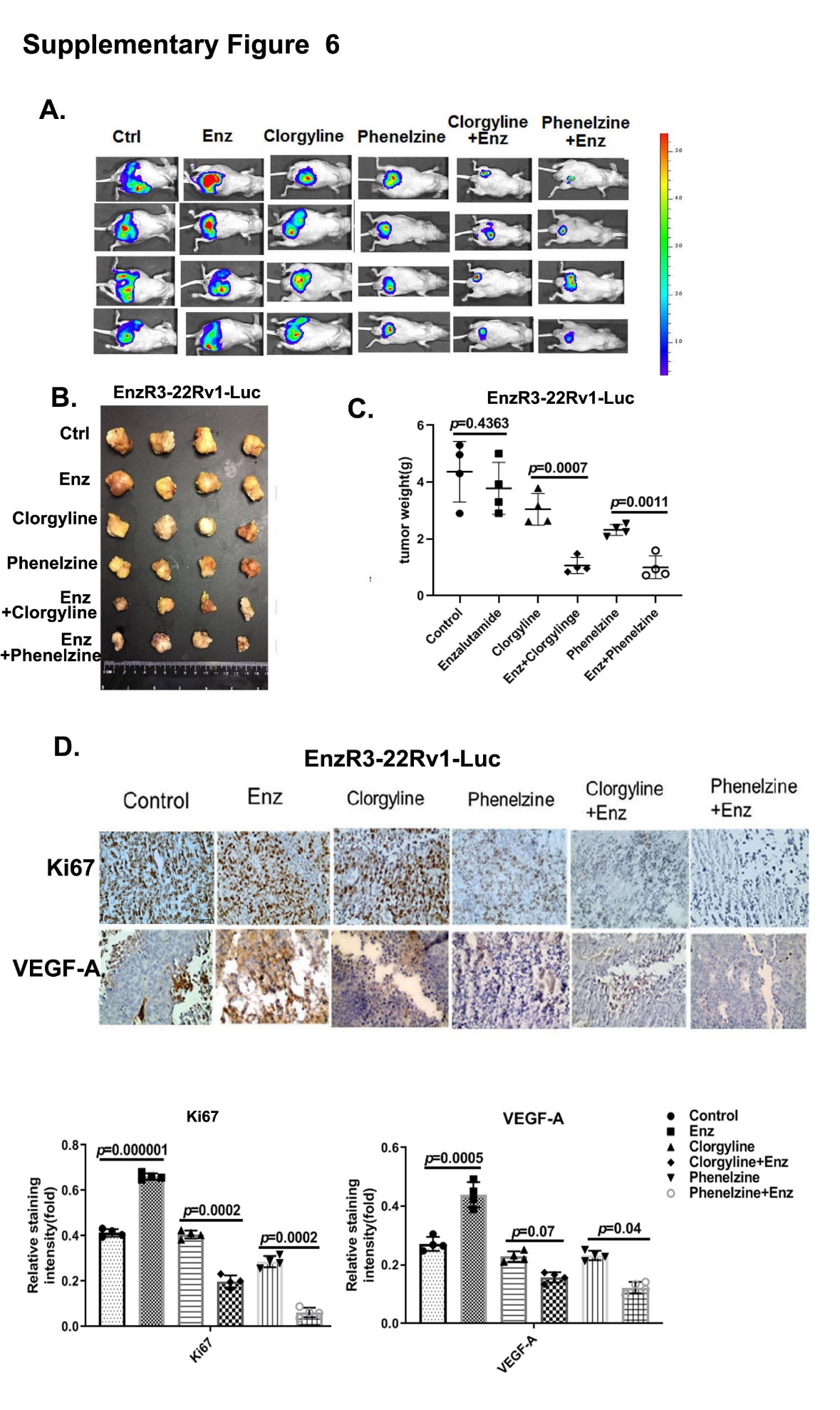
**

**Supplementary Figure 6**

(A) Mice implanted with EnzR3-22RV1-Luc xenografts (n=4) were treated with vehicle control, Enz (30 mg/kg), clorgyline (10 mg/kg), phenelzine (30 mg/kg), Enz+clorgyline (30 mg/kg+10 mg/kg), or Enz+phenelzine (30 mg/kg+30 mg/kg). The tumor sizes were monitored by In vivo imaging system (IVIS). (B-C) After sacrifice, tumors of the 6 groups were collected (B) and weighed (C). (D) IHC staining of Ki67 and VEGF-A in EnzR3-22Rv1-Luc tumors were performed. For D Quantitations are mean±SEM, P-value was determined by two-tailed paired t test.

**
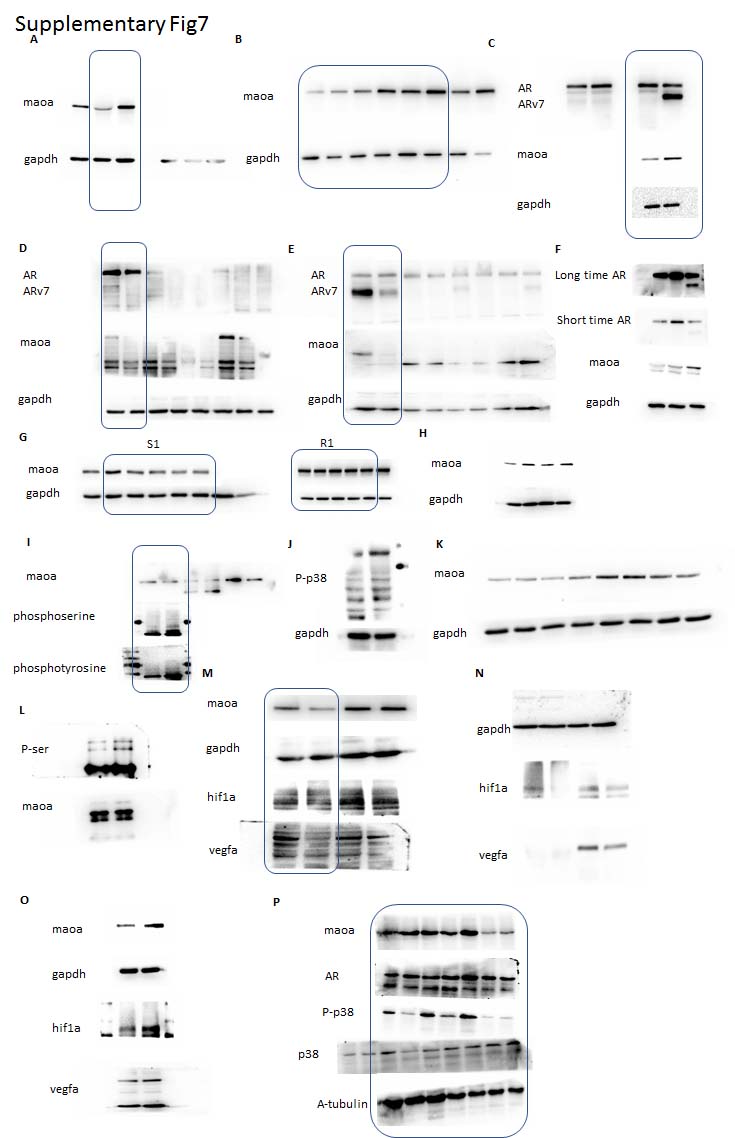
**

**Supplementary Figure 7**

(A) The raw blot of Fig. 1C. (B) The raw blot of Fig. 3C. (C) The raw blot of Fig. 3E. (D) The raw blot of Fig. 3F.(E) The raw blot of Fig. 3H. (F) The raw blot of Fig. 4H. (G) The raw blot of Fig. 4I. (H) The raw blot of Fig. 4J. (I) The raw blot of Fig. 4K. (J) The raw blot of Fig. 4L. (K) The raw blot of Fig. 4M. (L) The raw blot of Fig. 4N. (M) The raw blot of Fig. 5B. (N) The raw blot of Fig. 5C. (O) The raw blot of Fig. 5D. (P) The raw blot of Fig. 6A.

**
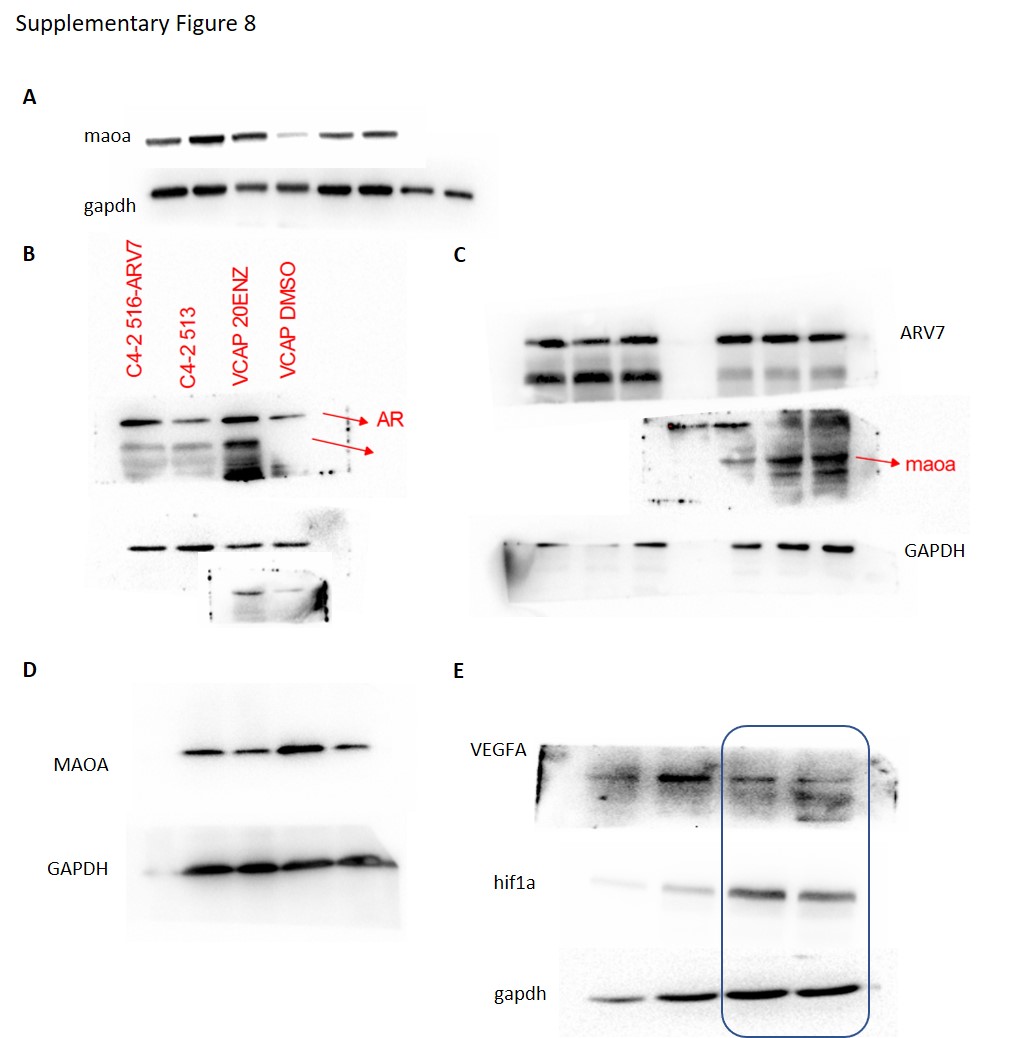
**

**Supplementary Figure 8**

(A) The raw blot of SFig. 1B. (B) The raw blot of SFig. 1C. (C) The raw blot of SFig. 3B. (D) The raw blot of SFig. 5A. (E) The raw blot of SFig. 5B.

**Supplementary Table 1**

| **Primer** | **Sequence** |
| --- | --- |
| MAO-A F | 5'-GAATCAAGAGAAGGCGAGTATCG-3' |
| MAO-A R | 5'-GGCAGCAGATAGTCCTGAAATG-3' |
| ARV7 F | 5'-CAGGGATGACTCTGGGAGAA-3' |
| ARV7 R | 5'-GCCCTCTAGAGCCCTCATTT-3' |
| GAPDH F | 5'-GCACCGTCAAGGCTGAGAAC-3' |
| GAPDH R | 5'-TGGTGAAGACGCCAGTGGA-3' |
| GLUT1 F | 5'-GGCCAAGAGTGTGCTAAAGAA-3' |
| GLUT1 R | 5'-ACAGCGTTGATGCCAGACAG-3' |
| N-cadherin F | 5'-TCAGGCGTCTGTAGAGGCTT-3' |
| N-cadherin R | 5'-ATGCACATCCTTCGATAAGACTG-3' |
| Timp F | 5'-CATGTGCAGTACATCCATACGG-3' |
| Timp R | 5'-CATCATAGACGCGACCTGTCA-3' |
| VEGF-A F | 5'-AGGGCAGAATCATCACGAAGT-3' |
| VEGF-A R | 5'-AGGGTCTCGATTGGATGGCA-3' |
| HIF-1a F | 5'-GTGTACCCTAACTAGCCGAGG-3' |
| HIF-1a R | 5'-GCAGTGCAATACCTTCCATGTT-3' |

**Supplementary Table 2**

| **Plasmid** | **Sequence** |
| --- | --- |
| shMAO-A-1 top | 5'-CCGGAGTGAGCGTCTCGTTCAATATGGATCCATATTGAACGAGACGCTC ACTTTTTTG-3' |
| shMAO-A-1 bottom | 5'-AATTCAAAAAAGTGAGCGTCTCGTTCAATATGGATCCATATTGAACGA  GACG CTCACT-3' |
| shMAO-A-2 top | 5'CCGGAGCAGAGAGAAACCAGTTAATGGATCCATTAACTGGTTTCTCTCTGCTTTTTTG 3' |
| shMAO-A-2 bottom | 5'-AATTCAAAAAAGCAGAGAGAAACCAGTTAATGGATCCATTAACTGGTTTCT C TCTGCT-3' |
| shARv7 top1 | 5'-CCGG AGGCTAATGAGGTTTATTT-3' |
| shARv7 bottom1 | 5'-GGATCC AAATAAACCTCATTAGCCT-3' |
| shARv7 top2 | 5'-GGATCC AAATAAACCTCATTAGCCT TTTTTG-3' |
| shARv7 bottom2 | 5’-AATTCAAAAA AGGCTAATGAGGTTTATTT-3' |
| pwpi MAO-A gibson F | 5'-GTGAGGAATTTCGACATTTAAATTTA ATG GAG AATCAAGAGAAG GC-3' |
| pwpi MAO-A gibson R | 5'-TCCTGCAGCCCGTAGTTTTCAAGACCGTGGCAGGAGCT-3'​ |
