# Supplementary material for: The MAO inhibitors phenelzine and clorgyline revert enzalutamide resistance in castration resistant prostate cancer
Source: Nat Commun. 2020 Jun 1;11:2689. doi: 10.1038/s41467-020-15396-5 (PMC7264333; doi:10.1038/s41467-020-15396-5)
Supplement: Supplementary file 2 — Reporting Summary [file 41467_2020_15396_MOESM2_ESM.pdf]

# nature research

Corresponding author(s): Chawnshang Chang

Last updated by author(s): 2020/02/16

# Reporting Summary

Nature Research wishes to improve the reproducibility of the work that we publish. This form provides structure for consistency and transparency in reporting. For further information on Nature Research policies, see Authors & Referees and the Editorial Policy Checklist .

## Statistics

For all statistical analyses, confirm that the following items are present in the figure legend, table legend, main text, or Methods section.

| n/a | Confirmed | |
|---|---|---|
| ☐ | ☒ | The exact sample size (*n*) for each experimental group/condition, given as a discrete number and unit of measurement |
| ☐ | ☒ | A statement on whether measurements were taken from distinct samples or whether the same sample was measured repeatedly |
| ☐ | ☒ | The statistical test(s) used AND whether they are one- or two-sided<br>*Only common tests should be described solely by name; describe more complex techniques in the Methods section.* |
| ☐ | ☒ | A description of all covariates tested |
| ☐ | ☒ | A description of any assumptions or corrections, such as tests of normality and adjustment for multiple comparisons |
| ☐ | ☒ | A full description of the statistical parameters including central tendency (e.g. means) or other basic estimates (e.g. regression coefficient) AND variation (e.g. standard deviation) or associated estimates of uncertainty (e.g. confidence intervals) |
| ☐ | ☒ | For null hypothesis testing, the test statistic (e.g. *F*, *t*, *r*) with confidence intervals, effect sizes, degrees of freedom and *P* value noted<br>*Give P values as exact values whenever suitable.* |
| ☒ | ☐ | For Bayesian analysis, information on the choice of priors and Markov chain Monte Carlo settings |
| ☒ | ☐ | For hierarchical and complex designs, identification of the appropriate level for tests and full reporting of outcomes |
| ☒ | ☐ | Estimates of effect sizes (e.g. Cohen's *d*, Pearson's *r*), indicating how they were calculated |

*Our web collection on statistics for biologists contains articles on many of the points above.*

## Software and code

Policy information about availability of computer code

| | |
|---|---|
| Data collection | We used the Bio-rad Image-lab 4.0.1 for collecting WB data. We used Bio-rad CFX manager 3.0 for collecting QPCR data. We used Perkin elmer In vivo imaging system (IVIS) system to collect the 22Rv1-luc tumors in vivo images |
| Data analysis | All of the statistical analysis were performed by GraphPad Prism 8.0.2. |

For manuscripts utilizing custom algorithms or software that are central to the research but not yet described in published literature, software must be made available to editors/reviewers. We strongly encourage code deposition in a community repository (e.g. GitHub). See the Nature Research guidelines for submitting code & software for further information.

## Data

Policy information about availability of data

All manuscripts must include a data availability statement. This statement should provide the following information, where applicable:

- Accession codes, unique identifiers, or web links for publicly available datasets
- A list of figures that have associated raw data
- A description of any restrictions on data availability

The data that support the findings of this study are available from the corresponding author upon reasonable request. The Supplementary Figure 4 data is based on the GEO dataset (GSE106559)

# Field-specific reporting

Please select the one below that is the best fit for your research. If you are not sure, read the appropriate sections before making your selection.

☒ Life sciences ☐ Behavioural & social sciences ☐ Ecological, evolutionary & environmental sciences

For a reference copy of the document with all sections, see nature.com/documents/nr-reporting-summary-flat.pdf

# Life sciences study design

All studies must disclose on these points even when the disclosure is negative.

| | |
|---|---|
| Sample size | The determination of the sample size is based on the bio-statistic analysis. Based on the appropriate sample size, the results can reach the statistical significance. P-value was determined by two-tailed paired t test. |
| Data exclusions | No data were excluded. |
| Replication | All of the experiments were repeated for at least 3 times. All the attempts were successful. |
| Randomization | We implanted the EnzR1-C4-2 or 22RV1 cells into the mice. After the tumor size reaching to 200mm3. We randomly separated the equal number of mice to each group for following experiments. For other in vitro xperiments, we didn't do the group comparison. |
| Blinding | The investigators were blinded to the group allocation. |

# Reporting for specific materials, systems and methods

We require information from authors about some types of materials, experimental systems and methods used in many studies. Here, indicate whether each material, system or method listed is relevant to your study. If you are not sure if a list item applies to your research, read the appropriate section before selecting a response.

## Materials & experimental systems

| n/a | Involved in the study | |
|---|---|---|
| ☐ | ☒ | Antibodies |
| ☐ | ☒ | Eukaryotic cell lines |
| ☒ | ☐ | Palaeontology |
| ☐ | ☒ | Animals and other organisms |
| ☒ | ☐ | Human research participants |
| ☒ | ☐ | Clinical data |

## Methods

| n/a | Involved in the study | |
|---|---|---|
| ☒ | ☐ | ChIP-seq |
| ☒ | ☐ | Flow cytometry |
| ☒ | ☐ | MRI-based neuroimaging |

## Antibodies

| | |
|---|---|
| Antibodies used | normal Rabbit IgG (sc-2027), AR (sc-816, N-20), GAPDH (sc-47724, 0411), tubulin (sc-23948, B-5-1-2), MAO-A (sc-271123, G-10), p38 (sc-81621, 9F12), VEGF-A (sc-7269, C-1), HIF1a (sc-13515, 28b) and ki67 (sc-23900, ki67) antibodies were from Santa Cruz Biotechnology, Inc (Santa Cruz, CA). Phosphoserine (ab9332)and phosphotyrosine (ab10321, PY20)antibodies were from Abcam, Inc (Cambridge, MA). p-p38 (#9211) antibody was from CST (Danvers, MA). Rabbit HRP-conjugated 2nd antibody (G21234) and mouse HRP-conjugated 2nd antibody(G21040) were from Invitrogen ( Carlsbad, CA). |
| Validation | All antibodies used in our study have been validated. Detailed information could be found on the manufactures' website as listed below. AR, MAO-A and HIIF1a antibodies have been validated by knocking down in C4-2 cells. VEGF-A antibody has been validated by knocking down HIF1a in C4-2 cells. p-p38 antibody has been validated by treating the C4-2 cells with p38 inhibitor. AR : https://www.scbt.com/p/ar-antibody-n-20, GAPDH: https://www.scbt.com/p/gapdh-antibody-0411, tubulin: https://www.scbt.com/p/alpha-tubulin-antibody-b-5-1-2, MAO-A: https://www.scbt.com/p/mao-a-antibody-g-10, p38: https://www.scbt.com/p/p38alpha-antibody-9f12, VEGF-A: https://www.scbt.com/p/vegf-antibody-c-1, HIF-1a: https://www.scbt.com/p/hif-1alpha-antibody-28b, Ki67: https://www.scbt.com/p/ki-67-antibody-ki-67, Phosphoserine:https://www.abcam.com/phosphoserine-antibody-ab9332.html, phosphotyrosine: https://www.abcam.com/phosphotyrosine-antibody-py20-ab10321.html. p-p38: https://www.cellsignal.com/products/primary-antibodies/phospho-p38-mapk-thr180-tyr182-antibody/9211 |

## Eukaryotic cell lines

Policy information about cell lines

| | |
|---|---|
| Cell line source(s) | C4-2B ( CRL-3315), PC-3 (CRL-1435), VCaP (CRL-2876, 293T (CRL-1573), C4-2(CRL-3314) and CWR22RV1 (CRL-2505) cell lines were purchased from the American Type Culture Collection (ATCC, Manassas, VA). The C4-2B-EnzR cells were gifts from Dr |

| | |
|---|---|
| | Allan Gao from UC Davis. The C4-2-EnzR cells were established by ourselves. |
| Authentication | C4-2, C4-2B, PC3 and CWR22RV1 cells have been authenticated by human STR profiling cell authentication through Genetic Testing Biotechnology corporation (Suzhou, China) |
| Mycoplasma contamination | All of the cell lines are negative for mycoplasma contamination. |
| Commonly misidentified lines (See ICLAC register) | None of the cells were listed in the ICLAC. |

## Animals and other organisms

Policy information about studies involving animals; ARRIVE guidelines recommended for reporting animal research

| | |
|---|---|
| Laboratory animals | We used 6-8 weeks, male Athymic Nude Mouse, SCID mouse and B-NDG mouse. |
| Wild animals | The study did not involve the wild animals |
| Field-collected samples | The study did not involve the field-collected samples |
| Ethics oversight | The university of Rochester Committee on Animal Resources (UCAR) and the Harbin Medical University have approved the study protocol |

Note that full information on the approval of the study protocol must also be provided in the manuscript.
